# An Integrated Kinetic Modeling Framework for Copyrolysis of Biomass and Plastic Waste

**DOI:** 10.1021/acsomega.5c09278

**Published:** 2025-11-25

**Authors:** Hui Liu, Hesham Alhumade, Ali Elkamel

**Affiliations:** † Department of Chemical Engineering, 52484 University of Pittsburgh Johnstown, Johnstown, Pennsylvania 15904, United States; ‡ Department of Chemical and Materials Engineering, Faculty of Engineering, King Abdulaziz University, Jeddah 22254, Saudi Arabia; § K. A. CARE Energy Research and Innovation Center, King Abdulaziz University, Jeddah 22254, Saudi Arabia; ∥ Department of Chemical Engineering, Khalifa University, Abu Dhabi 20000, United Arab Emirates; ⊥ Department of Chemical Engineering, 8430 University of Waterloo, Waterloo N2L 3G1, Ontario, Canada

## Abstract

Developing robust kinetic models for pyrolytic processes is challenging due to the complex properties of solid feedstock materials and their intricate reaction pathways. This study introduced a three-module modeling framework designed to provide a systematic approach for addressing these challenges. A kinetic model was developed to simulate the copyrolysis of red oak wood and polyethylene terephthalate (PET) at a 1:1 mass mixing ratio. In the first module, a parallel reaction mechanism was developed, and the corresponding kinetic parameters were initially estimated using the Friedman method with thermogravimetric (TGA) data and subsequently refined through a least-squares optimization method. A kinetic model using the parallel reaction mechanism was used to predict the conversion of the solid mixture. Due to the limitations of TGA data, the kinetic model was incapable of predicting the product yields from copyrolysis. In the second module, the kinetic model was retrained with experimental data of copyrolysis in a vertical-tube reactor, and the parallel reaction mechanism was also updated with the mass distribution coefficients of bioproducts calculated using the SLSQP (Sequential Least Squares Programming) method. However, this model exhibited inaccuracies at a higher temperature, 700 °C, indicating the absence of crucial secondary reactions. To address this issue, the kinetic model was restructured by combining tar decomposition reactions with the parallel-reaction mechanism. The kinetic parameters of tar decomposition reactions were calculated using the PSO (Particle Swarm Optimization) method. Finally, the kinetic model using the combined reaction mechanism successfully simulated copyrolysis and was validated with experimental data at 500, 600, and 700 °C, providing accurate predictions for both solid conversion and product generation. The findings of this work demonstrate that the methodology for identifying reaction mechanisms and determining kinetic parameters can also be valuable to the modeling of other complex pyrolytic processes.

## Graphical abstract

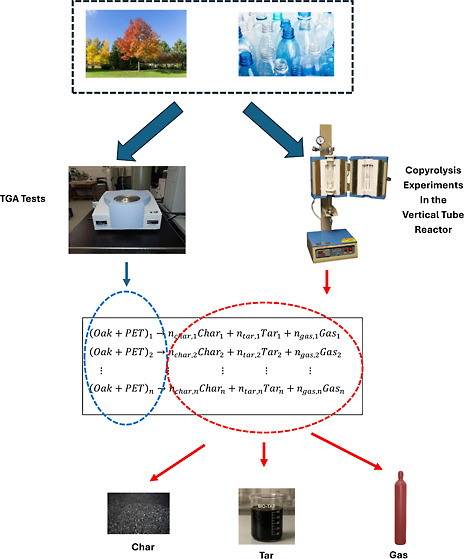

## 1 Introduction

Fossil fuels, such as petroleum oil, natural gas, and coal, are the world’s primary energy sources. However, the surging energy demand from industries has raised concerns regarding long-term energy sustainability.^1^ Additionally, the combustion of fossil fuels releases substantial amounts of waste gases including carbon dioxide, methane and nitrous oxide, contributing significantly to global warming and climate change.^2,3^ These emissions also pose health risks to communities due to exposure to pollutants.^4,5^ These concerns urge the exploration of cleaner and more sustainable energy sources.^6,7^

Biomass, a renewable energy source, can be considered as an alternative to fossil fuels.^8,9^ Through utilization technologies, biomass can be used to generate energy and produce valuable chemicals. Among these technologies, pyrolysis stands out as a promising approach. In the process, biomass is thermally decomposed into valuable chemicals without the presence of oxygen.^10–12^ Recently, researchers have shown increasing interest in the copyrolysis of biomass with plastic wastes.^13–15^ During copyrolysis, both biomass and plastic waste can be effectively converted into valuable chemicals, such as biochar, bio-oil (tar), and syngas.

Copyrolysis can offer several advantages over conventional pyrolysis of biomass. As discovered by the researchers,^16–18^ copyrolysis enhances the overall product yield, due to the synergistic interaction between biomass and plastics. Furthermore, copyrolysis improves the quality of bio-oil (tar) by lowering its oxygen content and increasing its calorific values. These improvements make bio-oil produced from copyrolysis more suitable feedstock for transportation fuel production.^19^

A thorough understanding of kinetics is crucial for designing and optimizing copyrolysis processes. Kinetic models incorporating proper reaction kinetics can provide accurate predictions in the production of bioproducts, including char, tar, and gas, from copyrolysis. The model predictions can then be utilized to design copyrolysis reactors and determine the optimal operating conditions for the reactors. However, the reaction kinetics of copyrolysis are complicated due to the complex chemical properties of biomass and plastics. Various kinetic models were previously developed to simulate biomass pyrolysis. They are mainly categorized into two groups: single-step and multistep kinetic models.^20–22^ Considering the complex properties of biomass, the multistep modeling approach is a more appropriate option. Furthermore, detailed information on the kinetic triplet including the pre-exponential factor (*A*), activation energy (Ea), and reaction mechanism (*f*(α)) for each reaction step is also required to complete the kinetic modeling.

For copyrolysis, kinetic modeling work was not reported as widely as that of biomass pyrolysis. Among these limited modeling studies, lumped kinetic modeling (LKM) is commonly used to reduce the model complexity while still delivering accurate predictions of products from copyrolysis. In this approach, multiple species are grouped into a small number of lumps, and each lump represents a class of chemical species with similar properties, such as boiling ranges.

Thuan et al.^23^ reported their lumped-kinetic modeling work on copyrolysis of palm kernel cake with polystyrene in a closed-tubing reactor. Three irreversible series and parallel reactions were developed to simulate copyrolysis in isothermal conditions. A least-squares curve fitting function was coupled with the kinetic model to predict two main products, including gas (noncondensable gases) and liquid (condensable volatiles). The reaction rate constants and reaction orders were varied during the optimization calculation to predict production of gas and liquid in the experiments at three temperature settings.

Aysan et al.^24^ presented their reaction schemes to simulate plastic pyrolysis. In their reaction schemes, three lumped products including char, tar, and gas were defined, and eight irreversible first-order chemical reactions were proposed. To improve modeling accuracy, a nonlinear least-squares method was applied to estimate kinetic parameters and predict production of char, tar, and gas at three experimental temperature settings. Sebastian-Mark et al.^25^ proposed several priori lumped-kinetic models to simulate copyrolysis of low-density polyethylene (LDPE) powder and a heavy petroleum fraction in a lab-scale tubular reactor. Eight lumps were defined, and three priori lumped reaction mechanisms, including the sequential, parallel, and combined LKMs, were developed to predict product yields. Each mechanism consisted of multiple irreversible and first-order reactions. A surrogate optimization model was integrated into the kinetic model to find optimal activation energies and achieve data-fit predictions across the reactor temperatures ranging from 400 to 500 °C. These reaction mechanisms were also utilized to predict the same process in a scaled-up pyrolysis reactor.

As described in previous studies, the multistep reaction mechanisms were employed to simulate copyrolysis. The reaction mechanisms were either assumed or adopted from the literature. They differ significantly from each other due to the variety of solid feedstock materials used in copyrolysis. These reaction mechanisms are unique and only valid for the materials specified in the studies and cannot be directly applied to other materials. As for the modeling of copyrolysis process, a new reaction mechanism is always required when a new feedstock material is selected. The modeling work remains challenging, because exploring appropriate reaction mechanisms for each solid material and identifying proper kinetic parameters for each stage of the process are both difficult and time-consuming.

Kinetic studies using isoconversional methods with TGA (thermogravimetric analysis) data can potentially provide a solution to this issue. Since TGA data are unique for each solid material, the reaction mechanism and kinetic parameters determined from the TGA data have the potential to provide more accurate model predictions for each individual solid material. TGA is a thermal analysis technique used to examine the pyrolytic behavior of solid samples by measuring mass loss over time under a controlled inert atmosphere. TGA tests are typically conducted multiple times at different heating rates. TGA data are generally used in kinetic studies with isoconversional methods to provide information about reaction mechanisms and estimate kinetic parameters. The isoconversional methods assume the reaction rate is a function of the reaction temperature at specific conversion levels. Based on this assumption, the kinetic parameters including pre-exponential factors and activation energies are calculated accordingly.^26–28^

Torres-Sciancalepore et al.^29^ conducted comparative kinetic and thermodynamic studies to investigate the pyrolysis behavior of quince waste (QW) and pectin-free quince waste (PFQW). The Flynn–Wall–Ozawa (FWO) isoconversional method was applied to calculate the activation energies. Additionally, the pre-exponential factors were calculated with the kinetic compensation effect, while the reaction mechanisms (*f*(α)) were determined by the master-plots method. In a separate study, Torres-Sciancalepore et al.^30^ conducted kinetic studies to describe the pyrolytic process of rosehip husk waste (RHW). The activation energies and pre-exponential factors were also calculated accordingly, which were further employed in another study on the synergistic effects of the mixing ratio between rosehip seed and husk wastes.^31^

Despite the potential of isoconversional methods coupled with TGA data, the issues of finding proper reaction mechanisms and kinetic parameters for individual samples are still unresolved. This is mainly because the assumption of isoconversional methods may not be strictly valid for the processes involving multistep reactions such as pyrolysis and copyrolysis. The kinetic parameters estimated from isoconversional methods are mostly used to illustrate the multistep behavior of pyrolytic processes and are not used directly in multistep reaction models.^32–34^ The values of these kinetic parameters need to be adjusted before they can be used in kinetic models.^35–37^

Furthermore, the information from TGA data is limited and inadequate for the kinetic modeling work. TGA tests can only provide data on the weight loss of solid samples and cannot provide information on char, tar, and gas production, which are essential to kinetic modeling for predicting product generation. Thus, in addition to the TGA data, the yield data of bioproducts from copyrolysis are also required to complete the modeling work.

In this work, a three-module modeling framework was proposed to provide a systematic modeling approach for addressing these challenges. A kinetic model was developed to simulate copyrolysis and was employed to predict the production profiles of char, tar, and gas from copyrolysis. To find the proper kinetic parameters for the kinetic model, a series of TGA experiments were conducted on solid mixture samples at various heating rates. The resulting TGA data were analyzed using the Friedman isoconversional method to estimate the kinetic parameters including pre-exponential factors and activation energies. Since the kinetic parameters could not be used directly in the kinetic model, a least-squares optimization model was developed to refine the kinetic parameters. A parallel-reaction mechanism was also formulated and incorporated into the kinetic model, and the optimal kinetic parameters were then assigned to each reaction step within the mechanism.

Due to the limitations of TGA data, the kinetic model trained solely with TGA data was incapable of predicting the yields of bioproducts from copyrolysis. To solve this problem, a series of copyrolysis experiments were conducted in a vertical-tube reactor to obtain yield data of char, tar, and gas at various temperature settings. The collected yield data were then used to retrain the kinetic model to predict production profiles of bioproducts. Moreover, another optimization model based on the sequential least-squares quadratic programming (SLQP) method was applied to calculate the mass distribution ratios of bioproducts for each parallel reaction step using the data from the vertical-tube reactor. To further enhance model accuracy, tar decomposition reactions were combined with the existing parallel-reaction mechanism, and the particle swarm optimization (PSO) method was applied to calculate the optimal activation energies for each tar decomposition reaction. The kinetic model, updated with the distribution coefficients of products and the combined reaction mechanism, was then applied to simulate the copyrolysis process.

It is worth noting that the processes in the TGA tests and the vertical-tube reactor experiments are the same copyrolysis. Both the TGA analyzer and the vertical-tube reactor can be considered copyrolysis reactors: the former is a micropyrolysis reactor, and the latter is a scaled-up copyrolysis reactor. In other words, the intrinsic reaction kinetics of the processes occurring in these two instruments are the same, which is why the two types of data can both be integrated into the kinetic model for copyrolysis. However, they serve different purposes: the TGA data provide information on solid decomposition, representing the reactant-side changes in the copyrolysis reactions, while the yield data of bioproducts from the vertical-tube reactor provide information on product generation, reflecting the product-side changes in the reactions. In this way, the kinetic model trained with both the reactant and product data can predict not only the decomposition of solid materials but also the yields of bioproducts from copyrolysis.

## 2 Sample Analysis in a TGA Analyzer and Copyrolysis in a Vertical Tube Reactor

### 2.1 Sample Preparation and Experimental Procedures of Thermogravimetric Analysis

In this study, sample mixtures of northern red oak wood and PET (polyethylene terephthalate) were prepared for copyrolysis. It is important to note that the copyrolysis process investigated in this study is classified as slow pyrolysis, which operates at relatively low heating rates. As reported by Alhulaybi and Dubdub,^38^ Choi et al.,^39^ and Jia et al.,^40^ the proximate analysis of PET shows a moisture content of 0–0.52%, a fixed carbon content of 0–9.37%, a volatile matter content of 88.23–94.69%, and an ash content of 0–11.25%. The ultimate analysis shows a carbon content of 60.97–64.26%, an oxygen content of 31.38–33.82%, a hydrogen content of 4.25–4.37%, and a nitrogen content of 0–0.03%. As indicated by Carrasco et al.^41^ and Hu et al.,^42^ northern red oak on a dry basis contains a volatile matter content of 78.20–81.22%, a fixed carbon content of 18.67–20.59%, and an ash content of 0.16–0.31%. According to the ultimate analysis, northern red oak contains 46.80–48.97% carbon, 43.91–44.20% oxygen, 6.33–6.90% hydrogen, and 0.72–2.10% nitrogen.

Northern red oak wood in this work was collected from the University of Pittsburgh at Johnstown campus. The branches were chipped, ground, and were sieved to approximately 100 μm. The samples were placed into a glass beaker and dried in an atmospheric oven at 105 °C for 48 h. The PET sample was obtained from water bottles collected from dumpsters. The bottles were washed with deionized water, chipped into small pieces less than 2 mm, and dried for 24 h before the TGA tests.

TGA tests were conducted on each of red oak and PET samples using a thermogravimetric analyzer (STA 6000 by PerkinElmer). Each sample at a mass of 10 mg was tested at heating rates of 10, 20, 30, and 40 °C/min. Additionally, a 1:1 mass ratio mixture of red oak and PET was tested under the same heating conditions. Several studies were previously conducted on various mass ratios such as 0.80:0.20, 0.70:0.30, 0.60:0.40, 0.50:0.50, 0.4:0.60, and 0.20:0.80.^43–46^ Some reports indicated that a 0.50:0.50 (1:1) ratio enhanced the yield of bio-oil.^47,48^ Nevertheless, the optimal ratio of mixed materials depends on the characteristics of each component. In this work, a ratio of 1:1 was chosen to ensure that both biomass and plastic waste feedstocks contribute equally to the copyrolysis process, allowing for the assessment of synergistic effects. It should be noted that this chosen ratio may not be optimal for the current copyrolysis process, and more extensive studies on the mixing ratio are beyond the current scope of kinetic modeling but would be worthwhile for future studies.

During the TGA tests, samples were placed in a ceramic sample holder and heated from 30 to 800 °C at 4 heating rates. Nitrogen gas was fed into the analyzer at 80 mL/min to maintain an inert environment.

### 2.2 Copyrolysis of Biomass and PET in a Vertical Tube Reactor

To further investigate the kinetics of copyrolysis, a series of experiments were conducted in a vertical furnace system as shown in Figure 1. The vertical tube-furnace from the MTI Corporation is made of quartz, with a diameter of 2 in. and a height of 5 in.. The furnace was heated using electrical heating, and the heating profile was programmed with an application installed on a laptop. The experiments conducted in the vertical-tube reactor were nonisothermal: the reactor was initially heated from ambient temperature at a rate of 10 °C/min. Once the target temperature was reached, the reactor was then held at that temperature for 60 min. Three target temperatures were defined as 500, 600, and 700 °C.

**Figure 1:**
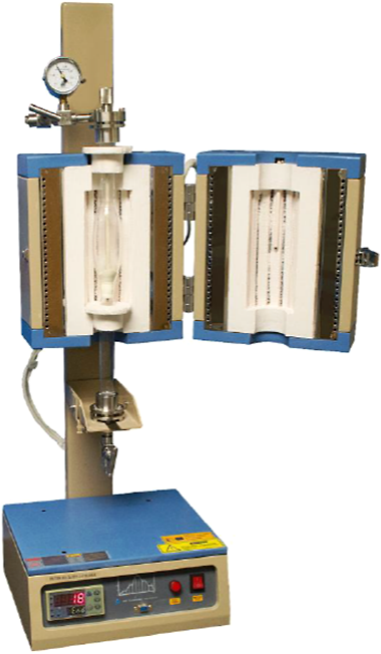
Vertical tube reactor for copyrolysis of red oak and PET samples.

In each experiment, 14 g of the sample mixture with a mass mixing ratio of 1:1 were placed in the vertical-tube reactor. Each material was chipped into pieces between 5 and 10 mm, which were larger than those used in the TGA tests. This choice was mainly driven by the goal of operating the reactor under conditions more representative of large-scale industrial production, where both equipment and operating costs are minimized. In industry, grinding solids into fine particles requires specialized equipment, which increases capital and production costs, especially at a large-scale production. Additionally, there are also safety and environmental concerns related to dust control and explosion hazards from fine particles. On the other hand, as reported by Van de Velden et al.^49^ and Brems et al.,^50^ heat transfer resistance within solid particles and mass transfer limitations, such as external and internal gas diffusion, play a significant role in reaction kinetics when large particles are used.

In the current work, we placed the solid mixture in the vertical-tube reactor for a period sufficiently long to ensure that the copyrolysis process was completed. This approach may help minimize the impact of mass and energy transfer limitations due to particle size. However, if more accurate transient predictions of product yields are required for the detailed design of a reactor, a CFD (computational fluid dynamics) simulation, in which mass transfer, energy transfer, and reaction kinetics are all considered, would be a more suitable approach than a kinetic model, as shown in our previous work.^51^

As illustrated in Figure 2, nitrogen gas was continuously fed to the vertical tube furnace to ensure an inert environment. The mixture samples were heated in the vertical tube furnace in the absence of oxygen gas. The mixture samples were gradually decomposed into char, tar, and gas. The tar vaporized due to the high temperature in the furnace and was sent together with noncondensable gases to the flasks in an icy water bath. The tar vapor condensed in the flasks and further condensed in a glass Graham condenser in which the icy water was circulated. The rest of the gas stream was finally collected as incondensable gas.

**Figure 2:**
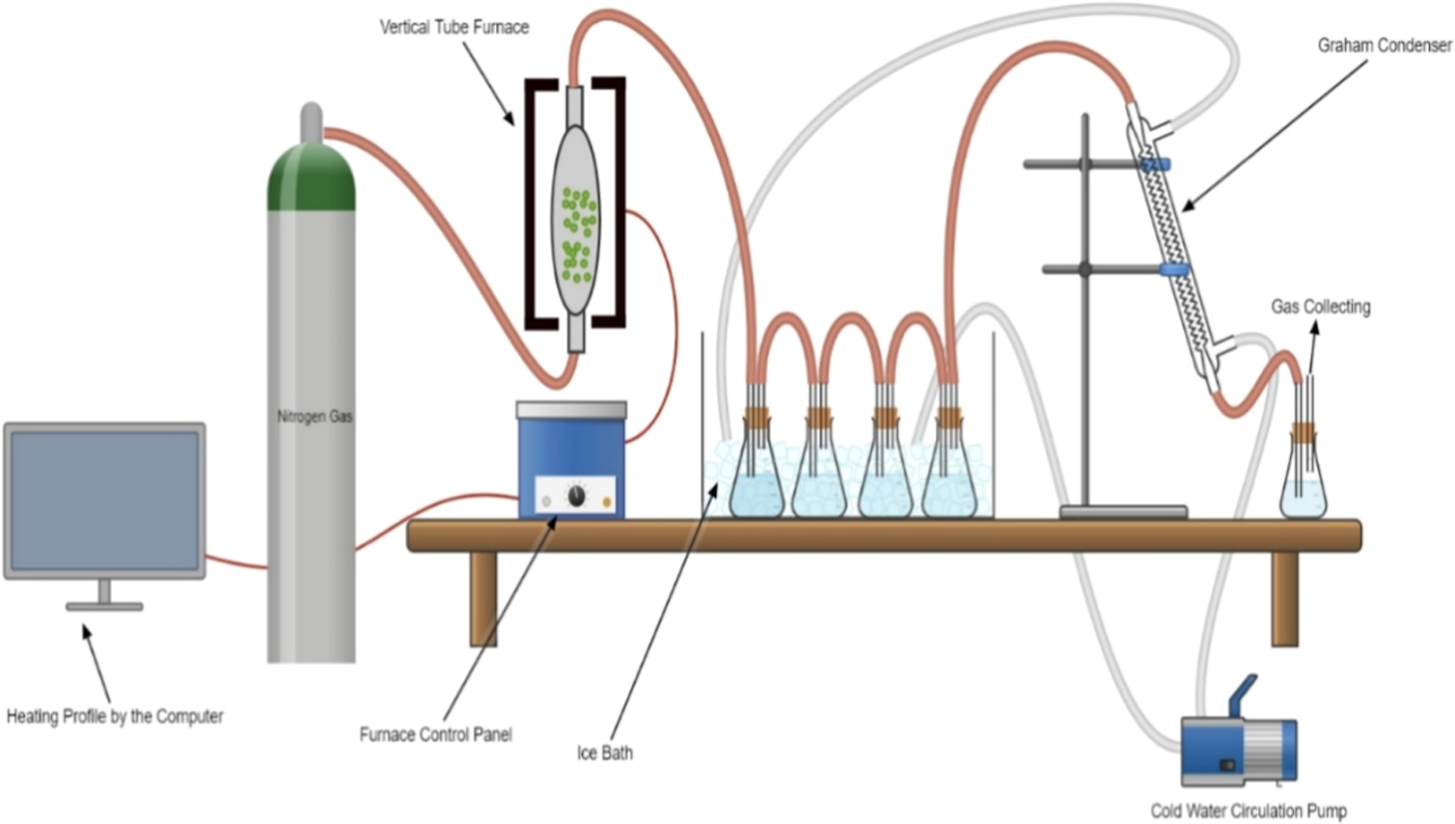
Experiment setup of Co-pyrolysis in the vertical tube reactor.

The weights of char, tar (condensable gases), and noncondensable gases were determined for each experiment. After each trial, the solid residue in the vertical tube furnace was weighed and recorded as the weight of char. The downstream components of the furnace system, located after the tube furnace, including the connection tubing, the Graham condenser, and other parts, were measured before and after each experiment. The change in weight was attributed to the weight of tar or condensable gases. The weight of noncondensable gases was calculated by subtracting the weights of solid residue and tar from the initial weight of the mixture sample.

## 3 The Kinetic Modeling Framework for Co-Pyrolysis of Biomass and Plastics

In this work, a modeling framework was proposed to develop a kinetic model to simulate copyrolysis of red oak wood and PET at a 1:1 mixing ratio.

The modeling framework comprised Modules 1, 2, and 3. In Module 1, a kinetic model using a parallel reaction mechanism was developed and trained with the TGA data to predict the conversion of red oak wood and PET samples at the 1:1 mixing ratio for copyrolysis. In Modules 2 and 3, the kinetic model was further trained with experimental data obtained from the vertical-tube reactor to predict the production profiles of bioproducts from copyrolysis. After being trained in Modules 1–3, the kinetic model was employed to predict both the conversion of reactants (solid mixture sample) and the production of products (char, tar, and gas) from copyrolysis.

Note that in the current work, the atomic balances between the reactants and products for each parallel reaction were not considered, and the reactants and products were categorized into lumped groups such as biomass, PET, char, tar, and gas to simplify the current modeling work. On the other hand, to build an accurate atomic balance for each parallel reaction, hundreds of chemicals in tar need to be analyzed, which is very challenging and beyond the capacity of our research facilities. Despite this limitation, the present modeling approach is still valuable, because it provides a framework that can be expanded to incorporate detailed reaction kinetics when detailed chemical characterization of bioproducts becomes available in future studies.

### 3.1 Reaction Kinetics Module 1

In Module 1, a kinetic model was built for the copyrolysis of red oak wood and PET at the 1:1 mixing ratio. The kinetic model was trained with the TGA data to predict the conversion of the solid mixture during copyrolysis. Meanwhile, a parallel reaction mechanism was developed and integrated with the kinetic model. This parallel reaction mechanism adopted the same assumptions as those of the Distributed Activation Energy Model (DAEM), a widely used approach in the kinetic modeling of biomass pyrolysis. The parallel reaction mechanism assumes that all parallel reactions are irreversible, independent, and follow the first-order kinetics, allowing for high computational efficiency and accurate predictions of multistage degradation behaviors.^52–56^

As illustrated in Figure 4, the TGA data, including the conversion (α) and decomposition rate (*r*) over the temperature, were utilized in an isoconversional method to calculate the pre-exponential factors, *A*, the activation energies, *E*_a_, for each reaction step, and the number of reaction steps, *n*. The calculated parameters were input into the kinetic model using the parallel reaction mechanism to simulate the decomposition of the mixture samples for copyrolysis in the thermogravimetric analyzer. An optimization method using the least-squares method was coupled with the kinetic model to calculate the optimal activation energies for each reaction step which were used in Modules 2 and 3.

**Figure 3:**
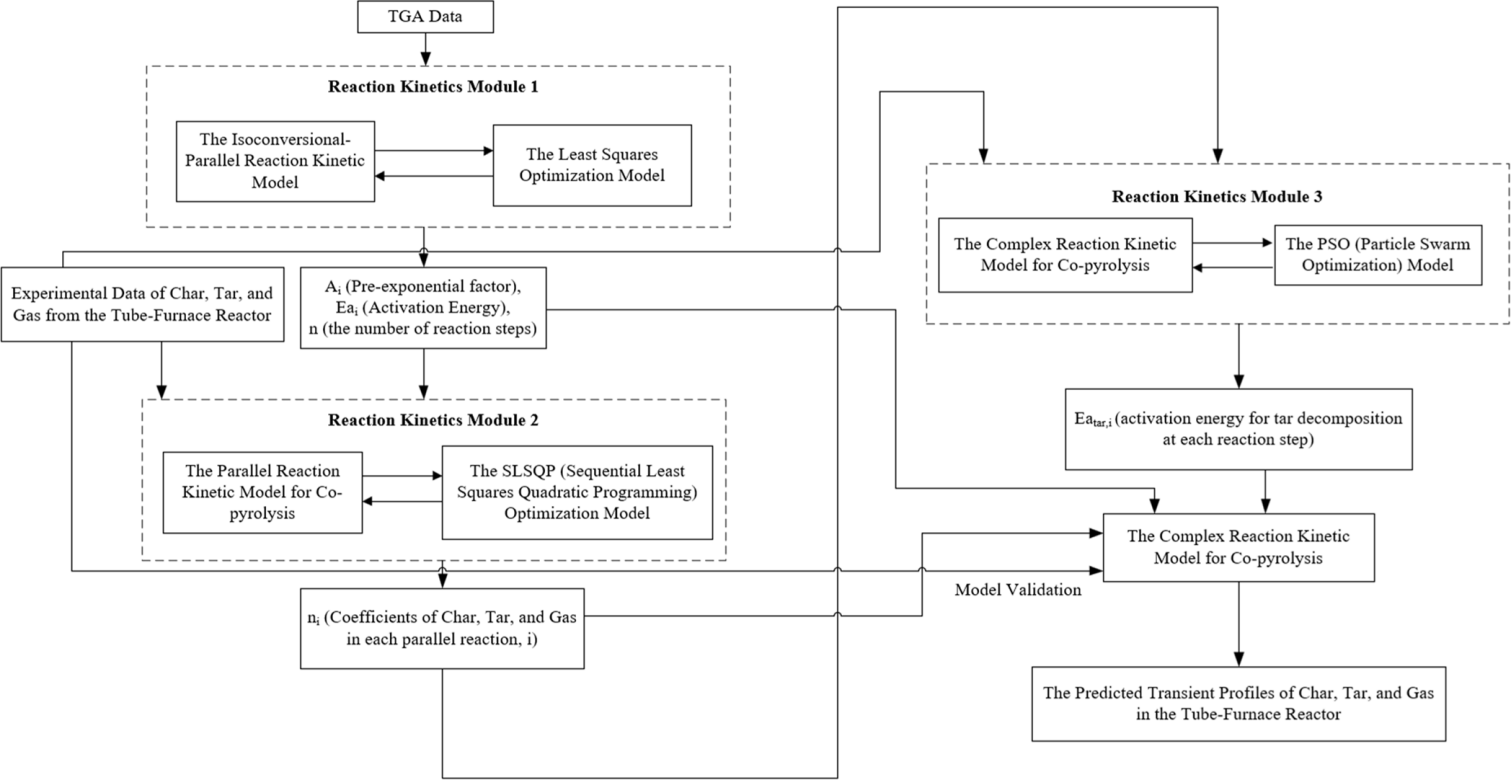
Kinetic modeling framework for co-pyrolysis in the vertical tube reactor.

**Figure 4:**
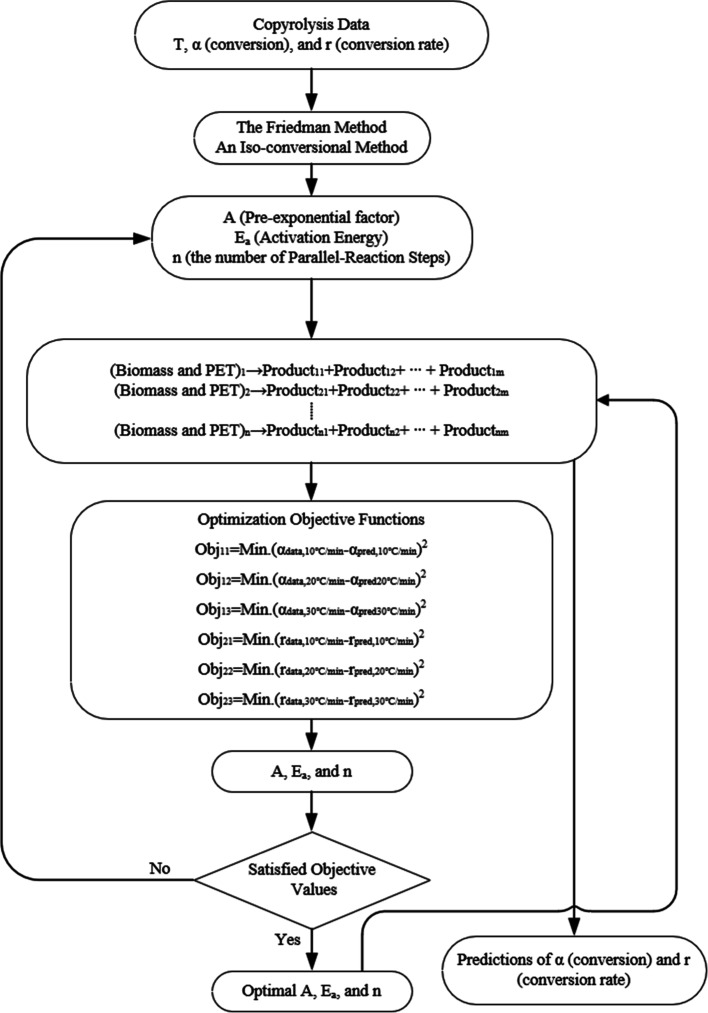
Chemical reaction module 1—the parallel reaction kinetic model scheme.

The conversion is calculated as follows

1 $$\alpha =\frac{m_{0}-m}{m_{0}-m_{f}}$$

where *m*_0_ is the initial mass of sample, *m*_f_ is the final mass of sample, *m* is the mass of sample at a time of *t*.

The iso-conversion method used in Module 1 is the Friedman method. This method was chosen because both the pre-exponential factor and activation energy can be calculated with the assumption of the first-order reaction for each parallel reaction step, and it has been proved to be well-integrated with the proposed parallel reaction mechanism in our previous work.^52,53^

The Friedman method is as shown below

2 $$\ln\left(\frac{d\alpha }{dt}\right)=\ln\left(Af\left(\alpha \right)\right)-\frac{E_{a}}{RT}$$

For the first-order reaction

3 f(α)=(1−α)

In the iso-conversional method, $\ln\left(\frac{d\alpha }{dt}\right)$ and $\frac{1}{T}$ are considered as a dependent variable and an independent variable, respectively. The term of ln­(Af­(α)) is considered the y intercept of the linear function. By assuming that the reaction is the first order, *A* can then be calculated with the achieved interceptive value.

The parallel reaction mechanism was defined as follows
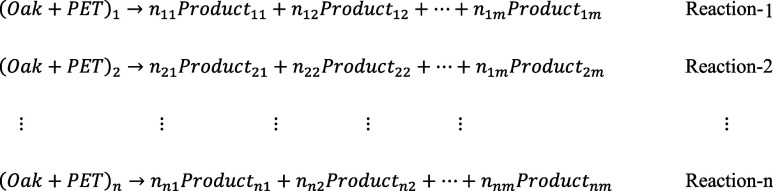


The conversion of biomass and plastics mixture is calculated in the following equations

4 $$\frac{d\alpha }{dt}=\frac{d\alpha_{1}}{dt}+\frac{d\alpha_{2}}{dt}+...+\frac{d\alpha_{n}}{dt}$$

5 $$\frac{d\left(\alpha_{n}\right)}{dt}=k_{n}\left(1-\alpha_{n}\right)$$

6 $$k_{n}=A_{n}\exp\left(-\frac{E_{a,n}}{RT}\right)$$

7 $$\alpha_{overall}=\sum_{i=1}^{n}\alpha_{i}$$

The objective of optimization model is to minimize the error from the predictions of α and $\frac{d\alpha }{dt}$ for the TGA experiments at 10, 20, and 30 °C/min.

The Least Squares Optimization Model is defined with 6 objective functions as follows

objective11=Min.(αdata,10⁢ C°/minute−αpred,10⁢ C°/minute)2 8

objective12=Min.(αdata,20⁢ C°/minute−αpred,20⁢ C°/minute)2 9

objective13=Min.(αdata,30⁢ C°/minute−αpred,30⁢ C°/minute)2 10

objective21=Min.(dαdtdata,10⁢ C°/minute−dαdtpred,10⁢ C°/minute)2 11

objective22=Min.(dαdtdata,20⁢ C°/minute−dαdtpred,20⁢ C°/minute)2 12

objective23=Min.(dαdtdata,30⁢ C°/minute−dαdtpred,30⁢ C°/minute)2 13

***Subject to***.

Equations 4–7.

***Control Variables:***

*E*_a_, the activation energies of parallel reaction steps.

The kinetic model in Module 1 was developed in Python, and the optimization model was solved using SciPy’s least-squares method. The computation time for Module 1 was approximately 6 h on a computer workstation with a CPU of i9–13900k and a GPU of RTX 4090.

### 3.2 Reaction Kinetics Module 2

In Module 2, the kinetic model was further trained with experimental data obtained from the vertical-tube reactor. The kinetic model was employed to predict the yields of bioproducts from the copyrolysis of the red oak wood and PET mixture.

As shown in Figure 3, an optimization model using the SLSQP (Sequential Least Squares Programming) method was applied to calculate the mass distribution coefficients of bioproducts in each parallel reaction step. The optimal mass distribution coefficients were then assigned to each reaction step in the kinetic model to predict the yields of char, tar, and gas from copyrolysis. In Module 2, the mass distribution coefficients of bioproducts were calculated using the parallel-reaction mechanism in which tar decomposition reactions were not considered.

However, tar decomposition was observed during the copyrolysis experiments in the vertical-tube reactor at temperatures above 600 °C. In terms of the modeling work, if tar decomposition reactions were added to the existing parallel-reaction mechanism, the reaction mechanism would become a complex reaction mechanism, which is a combination of parallel and series reactions. Calculating the mass distribution coefficients of bioproducts from such a complex reaction mechanism can be challenging.

To simplify the modeling work, an assumption was made that tar decomposition reactions only happen to a very small extent at relatively low temperature settings such as 500 and 600 °C, which is consistent with our findings in the experiments. Consequently, the kinetic model using the parallel reaction mechanism can still be valid for copyrolysis at such temperature settings, even though tar decomposition reactions are not considered. In this way, the mass distribution coefficients of bioproducts were calculated more efficiently with reasonable accuracy. As a result, in Module 2, the kinetic model was only trained with experimental data at the temperature settings of 500 and 600 °C.

As for the parallel reaction mechanism, the term of *Product*_*ij*_ previously defined in Reactions 1-n was replaced with specific bioproducts such as char, tar, and gas. The parallel reaction mechanism in Module 2 is shown as follows:
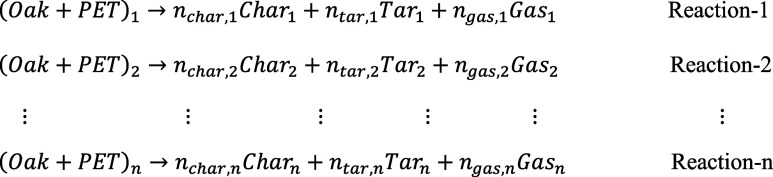


Assuming that each parallel reaction is an elementary reaction and follows the first order, the concentration of the mixture of biomass and PET, as a reactant, is calculated in the following equation

14 $$\frac{d{\left(Oak+PET\right)}_{i}}{dt}=-k_{i}{\left(Oak+PET\right)}_{i}$$

Char, tar, and gas in each parallel reaction as products are calculated as follows

15 $$\frac{d{\left(Char\right)}_{i}}{dt}=n_{char,i}k_{i}{\left(Oak+PET\right)}_{i}$$

16 $$\frac{d{\left(Tar\right)}_{i}}{dt}=n_{tar,i}k_{i}{\left(Oak+PET\right)}_{i}$$

17 $$\frac{d{\left(Gas\right)}_{i}}{dt}=n_{gas,i}{k_{i}\left(Oak+PET\right)}_{i}$$

The sum of the mass distribution coefficients of products is defined as 1.0 to maintain the mass balance for each parallel reaction.

18 $$n_{gas,i}+n_{tar,i}+n_{gas,i}=1.0$$

The total amounts of reactants and products from all parallel reactions are calculated as the overall consumption of the mixture of biomass and PET and the overall production of each bioproduct during copyrolysis.

19 $${\left(Oak+PET\right)}_{total}=\sum {\left(Oak+PET\right)}_{i}$$

20 $${Tar}_{total}=\sum {Tar}_{i}$$

21 $${Gas}_{total}=\sum {Gas}_{i}$$

where *k* is the reaction rate constant, *i* is the reaction-step number, and *n*_i_ is the mass distribution ratio of each bioproduct in the ith parallel reaction.

The optimization model in this module is shown as follows:

***Objective function***

Min.(Charpred,500⁢ C°−Chardata,500⁢ C°)2+(Tarpred,500 C°−Tardata,500 C°)2+(Gaspred,500 C°−Gasdata,500 C°)2+(Charpred,600 C°−Chardata,500 C°)2+(Tarpred,600 C°−Tardata,600 C°)2+(Gaspred,600 C°−Gasdata,600 C°)2

***Subject to***.

Equations 14–21.

***Control Variables***:*n*_i_, the mass-based coefficients of char, tar, and gas from each reaction step.

Module 2 was developed in Python, and the optimization model was calculated using the SLSQP method with the “minimize” solver in the SciPy library.

Note that in this work the calculations of product distribution coefficients are not bound by the atomic balances of the reaction steps and are only calculated based on experimental data at the temperature settings of 500 and 600 °C. The model accuracy can be improved significantly when detailed analysis data of chemical compounds in tar from copyrolysis become available in the future.

### 3.3 Reaction Kinetics Module 3

As mentioned previously, tar decomposition reactions were not considered in Module 2 for the simplification of the modeling work. However, if the reactor temperature is over 600 °C, the model accuracy can be significantly affected because tar decomposes rapidly at high temperatures and must be considered.

To address this issue, tar decomposition reactions were included in Module 3 and combined with the existing parallel reaction mechanism to complete the modeling work. The final version of the kinetic model using the combined reaction mechanism was retrained with experimental data at all three temperature settings, 500, 600, and 700 °C. Meanwhile, the mass distribution coefficients previously assigned to each parallel-reaction step in Module 2 were still applied to the same parallel-reaction steps in the combined mechanism. The kinetic model with the combined reaction mechanism was then used to predict the yields of char, tar, and gas from the copyrolysis of red oak wood and PET at a 1:1 mixing ratio.

The combined reaction mechanism was shown as follows
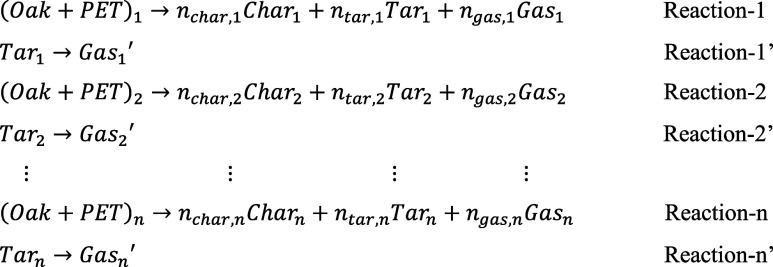


The biomass-PET mixture and char concentrations are still calculated from eqs 14 and 15, respectively. However, due to the tar decomposition, tar and gas concentrations are calculated as follows

22 $$\frac{d{\left(Tar\right)}_{i}}{dt}=n_{tar,i}k_{i}{\left(Oak+PET\right)}_{i}-k_{tar,i}^{\prime }{Tar}_{i}$$

23 $$\frac{d{\left(Gas\right)}_{i}}{dt}=n_{gas,i}{k_{i}\left(Oak+PET\right)}_{i}+k_{tar,i}^{\prime }{Tar}_{i}$$

where *k*′ is the reaction rate constant of tar decomposition.

The optimization model in Module 3 is shown as follows:

***Objective function***

Min.(Charpred,500 C°−Chardata,500 C°)2+(Tarpred,500 C°−Tardata,500 C°)2+(Gaspred,500 C°−Gasdata,500 C°)2+(Charpred,600 C°−Chardata,500 C°)2+(Tarpred,600 C°−Tardata,600 C°)2+(Gaspred,600 C°−Gasdata,600 C°)2+(Charpred,700 C°−Chardata,700 C°)2+(Tarpred,700 C°−Tardata,700 C°)2+(Gaspred,700 C°−Gasdata,700 C°)2

***Subject to***.

Equations 14,15,22, and 23.

***Control Variables:*** *E*_a,tar_, the activation energies of tar decomposition at each parallel reaction step.

The optimization model in Module 3 was calculated using the PSO (Particle Swarm Optimization) method from the Pymoo library with default settings. It took 4 h and 16 min to complete the computation for Module 3 on the computer workstation.

## 4 Results and Discussion

### 4.1 TGA Results of Three Samples (Red Oak, PET, and Mixture)

The conversion profiles of three samples were demonstrated in Figures 5–7. As shown in the figures, pyrolysis of the red oak sample started at around 100 °C and ended at around 750 °C. On the other hand, PET pyrolysis occurred in a very narrow temperature range, between 300 and 600 °C. The profile of copyrolysis is a transitional profile between those of the red oak and PET samples. It was observed that the conversion profiles were shifted to higher temperatures with increasing heating rate across the profiles of all samples (red oak, PET, and the mixture of red oak and PET).

**Figure 5:**
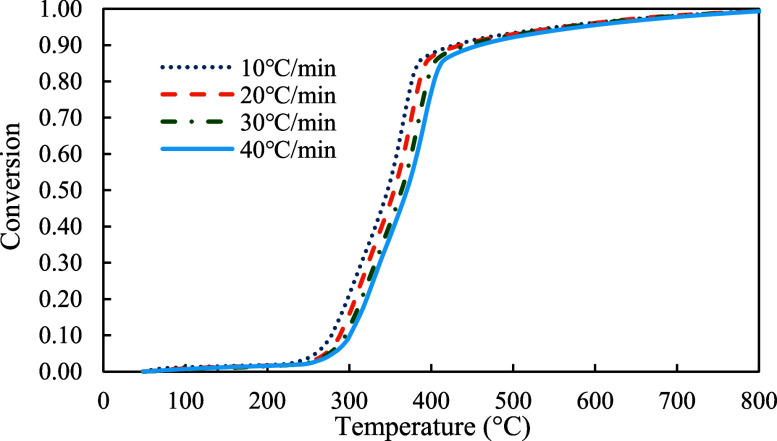
Conversion profiles of red oak at 10, 20, 30, and 40 °C/min.

**6:**
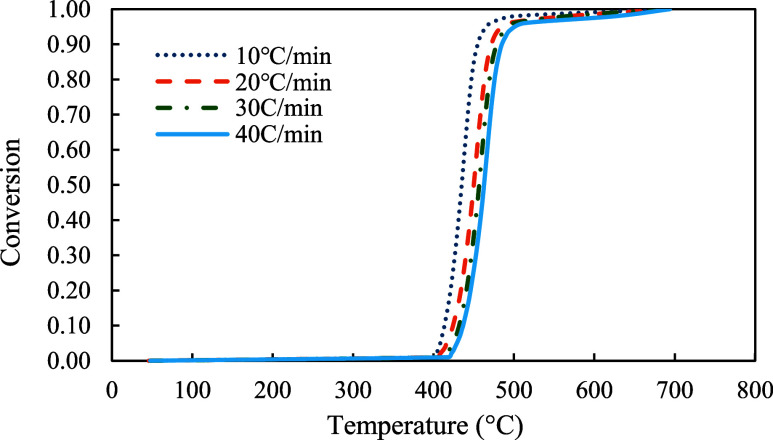
Conversion profiles of PET at 10, 20, 30, and 40 °C/min.

**Figure 7:**
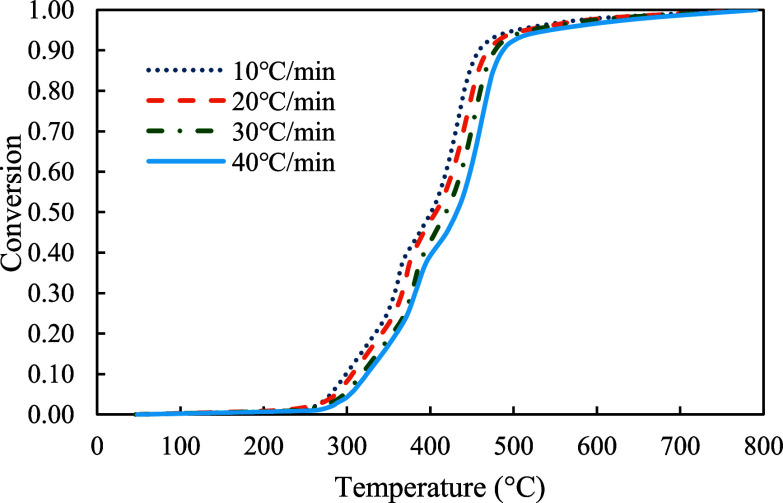
Conversion profiles of red oak and PET (1:1) at 10, 20, 30, and 40 °C/min.

The profiles of conversion rates were shown in Figures 8–10. The decomposition rate of red oak showed three peak points indicating the decomposition of hemicellulose, cellulose, and lignin. In comparison, the profile of PET sample only showed a single peak rate. The profile of copyrolysis showed three peak rates, similar to that of the red oak sample. It was also found that the decomposition rates increased with heating rates, indicating that a higher heating rate promotes a faster decomposition process.

**Figure 8:**
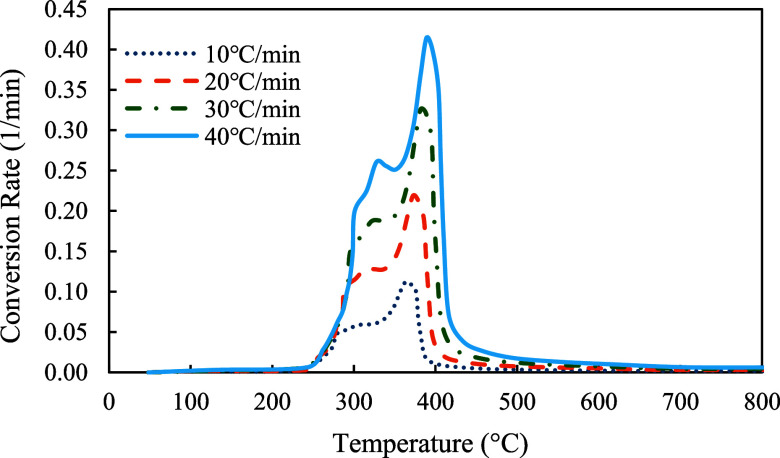
Conversion rates profiles of red oak at 10, 20, 30, and 40 °C/min.

**9:**
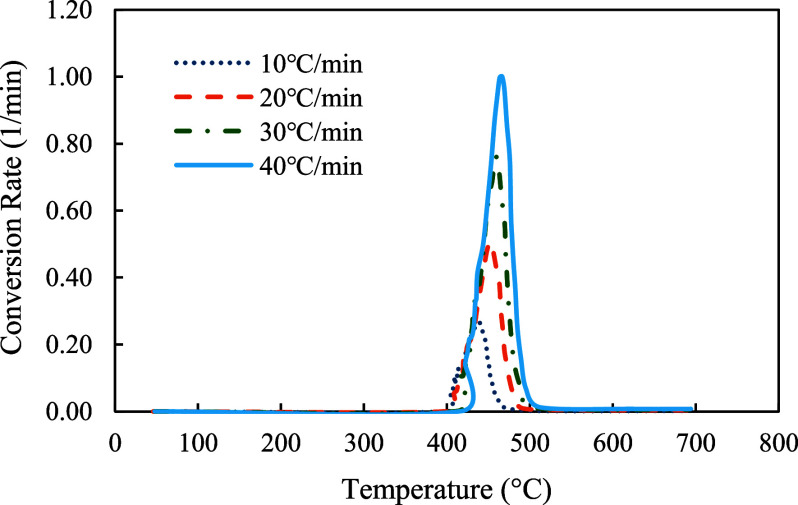
Conversion rates profiles of PET at 10, 20, 30, and 40 °C/min.

**Figure 10:**
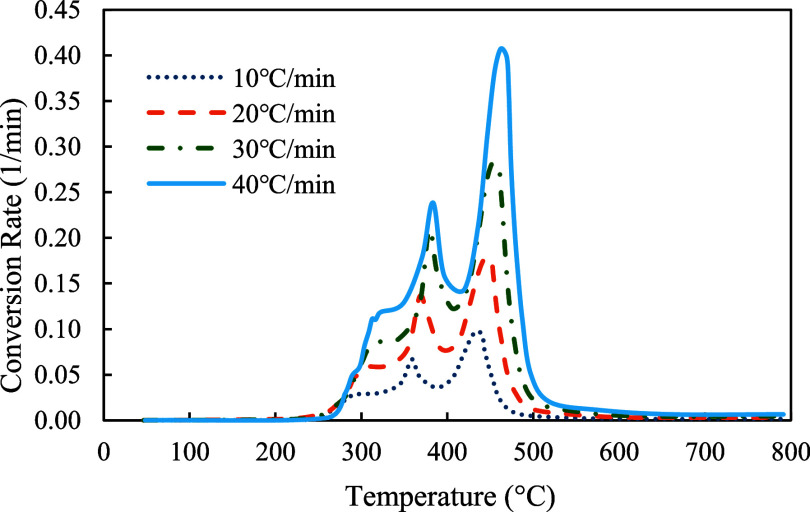
Conversion rates profiles of red oak and PET (1:1) at 10, 20, 30, and 40 °C/min.

To further demonstrate the differences between pyrolysis of each sample, the conversion profiles were compared in Figure 11. It was observed that pyrolysis of the red oak started at a lower temperature, while PET pyrolysis started at a higher temperature. The copyrolysis started at a temperature between those of red oak and PET samples. As illustrated in Figure 12, the decomposition rate of copyrolysis is smaller than that of red oak and PET at the corresponding temperatures.

**Figure 11:**
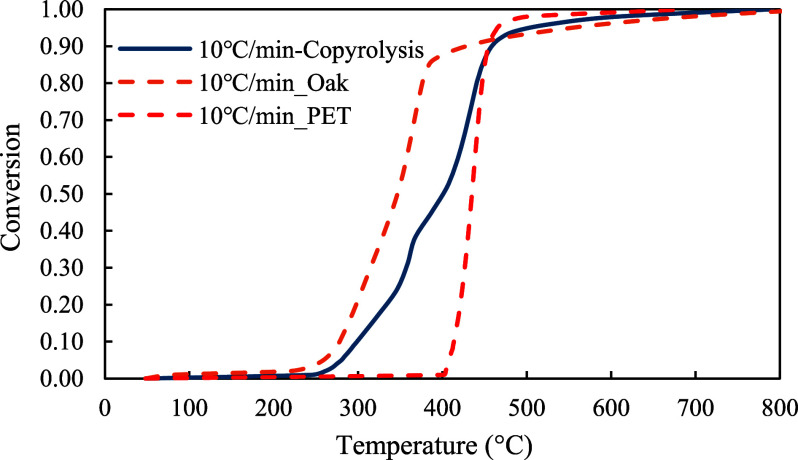
Conversion comparison of red oak, PET, and red oak &PET (1:1) at 10 °C/min.

**Figure 12:**
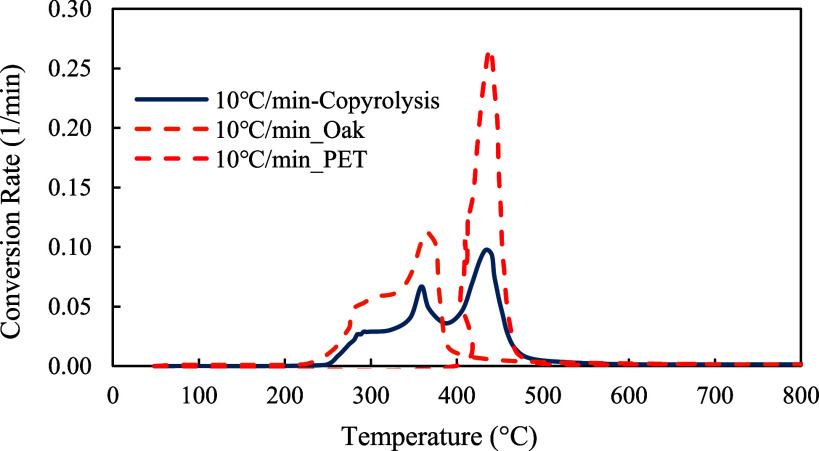
Conversion rate comparison of red oak, PET, and red oak &PET (1:1) at 10 °C/min.

To further investigate the synergistic effect of copyrolysis, the experimental data of decomposition rate were compared with the calculated profile, which was defined as follows

24 $${rate}_{calulated}={rate}_{redoak}\times \eta_{redoak}+{rate}_{PET}\times \eta_{PET}$$

25 $$\eta_{redoak}+\eta_{PET}=1.0$$

where rate_redoak_ and rate_PET_ are the decomposition rates of red oak and PET, respectively. η_redoak_ and η_PET_ are the mass fractions of red oak and PET in the mixture, respectively. The sum of these two terms is equal to 1.0.

The calculated rate, rate_calculated_, is the arithmetic average of the decomposition rates of red oak and PET samples based on the 1:1 mass mixing ratio, without considering the synergistic effect from copyrolysis. As demonstrated in Figure 13a–d, the decomposition rates of copyrolysis data are higher than the calculated profiles between 200 and 400 °C at all four heating rates, indicating the existence of synergistic effects. A lower experimental decomposition rate was observed afterward between 400 and 500 °C, because there were fewer remaining samples due to the high decomposition rate at the previous peak.

**Figure 13:**
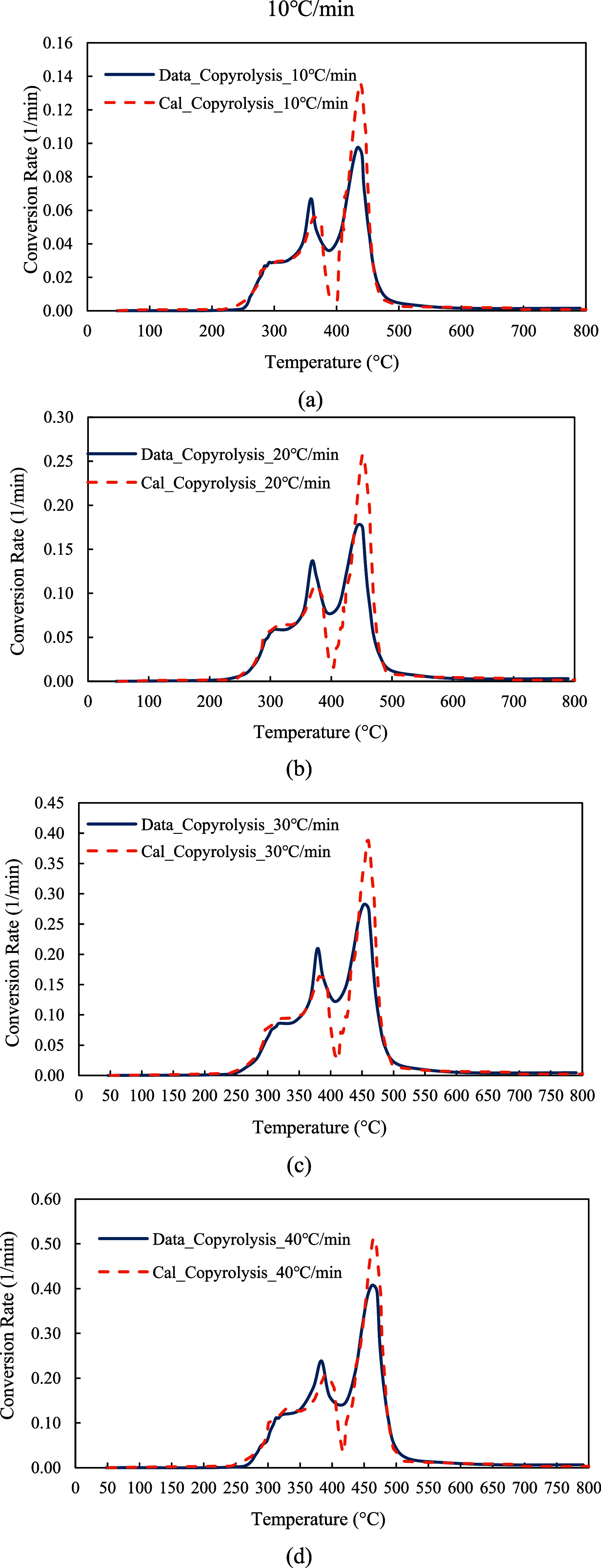
(a–d): Theoretical and experimental rate comparison at heating rates of 10–40 °C/min.

### 4.2 Experimental Results of Copyrolysis of Mixture (Biomass and PET) Samples in a Vertical Tube Reactor

The contents of char, tar, and gas from the experiments at 500, 600, and 700 °C are shown in Figure 14. It was observed that the char content remained relatively constant from 500 to 700 °C. The gas content was stable between 500 and 600 °C but increased significantly from 600 to 700 °C. The tar content gradually increased and reached a maximum from 500 to 600 °C; however, it dropped dramatically when the temperature was increased to 700 °C. The respective decrease in tar and increase in gas indicated the decomposition process of tar from 600 to 700 °C in which large molecules of tar were decomposed into small molecules of gases. It is why the tar decomposition for each parallel reaction needs to be included in Module 3. Again, it is worth noting that these production data were achieved in three nonisothermal experiments with three specific maximum temperatures.

**Figure 14:**
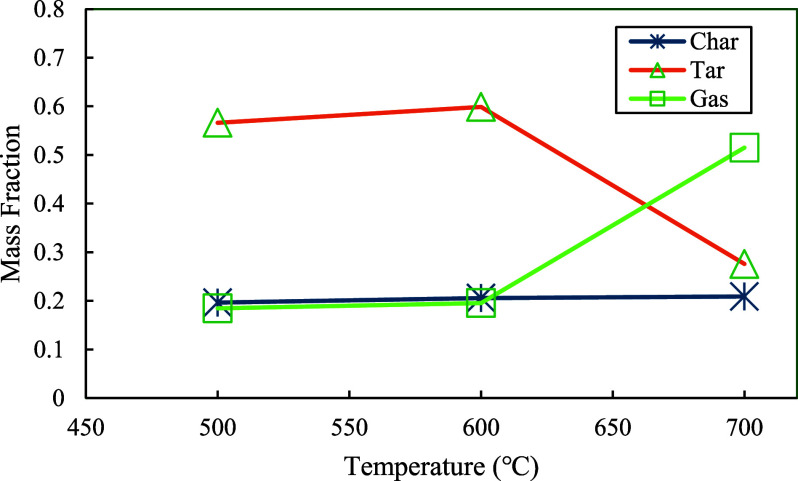
Experimental data of char, tar, and gas at the settings of 500, 600, and 700 °C.

Wu et al.^57^ also discovered similar trends of product yields from their copyrolysis experiments on corn stover (CS) and polypropylene (PP). As shown in their studies, at the mixing ratio of 1:1, the yield of tar increased until the temperature reached 550 °C and then decreased as the temperature continued to increase; meanwhile, the gas yield increased sharply as the temperature increased beyond 550 °C, while the char yield remained almost unchanged. It was concluded that the changes in the tar and gas yields were caused by the secondary pyrolytic cracking process.

### 4.3 Simulation Results of the Kinetic Model for TGA Tests and Vertical-Tube Reactor Copyrolysis

In Module 1, the TGA data at three heating rates (10, 20, and 30 °C/min) were analyzed using the Friedman method to estimate the kinetic parameters, including the pre-exponential factors and activation energies for the parallel-reaction mechanism. The kinetic parameters were then refined through a least-squares optimization model and assigned to each parallel reaction. The kinetic model was employed to predict the conversion (α) and conversion rate ( $\frac{d\alpha }{dt}$ ) of the red oak wood and PET mixture at a 1:1 mixing ratio. The model’s predictions were compared with TGA data at all four heating rates, including 10, 20, 30, and 40 °C/min, to validate the model.

The values of *A* and *E*_a_ estimated from the Friedman method were listed in Table 1. The values of *A* were applied directly to each parallel reaction step, while the values of *E*_a_ were refined in the optimization model and were shown as the optimal *E*_a_ in the table. It was observed that the activation energies were significantly optimized from the initially estimated values, ranging from 0.15 to 34.65%. The predicted α and $\frac{d\alpha }{dt}$ from the kinetic model using the optimal kinetic parameters were demonstrated in Figures 15a–d and 16a–d. As shown in the figures, the kinetic model presented accurate solid conversion profiles in the temperature range of 20–800 °C, and the predictions closely matched each conversion data point at all four heating rates. Additionally, the kinetic model accurately predicted the conversion rates, and the three-peak rates of characteristic features were successfully captured.

**Table 1:** Optimal A and *E*_a_ for the Parallel-Reaction Mechanism

reaction no.	A (1/min)	initial *E*_a_(kJ/mol)	optimal *E*_a_(kJ/mol)	difference *E*_a_ (%)
1	2.13 × 10^11^	136	170	24.73
2	6.88 × 10^9^	121	92.1	23.88
3	2.08 × 10^11^	137	177	29.61
4	3.60 × 10^12^	150	178	18.74
5	9.97 × 10^10^	133	152	13.83
6	6.70 × 10^12^	154	181	17.48
7	8.69 × 10^11^	145	153	5.34
8	1.91 × 10^12^	149	156	4.66
9	7.06 × 10^12^	157	181	15.77
10	3.01 × 10^12^	158	213	34.65
11	1.18 × 10^13^	170	201	18.44
12	6.93 × 10^14^	191	205	6.92
13	7.23 × 10^14^	195	182	6.65
14	1.19 × 10^16^	217	242	11.61
15	8.87 × 10^17^	246	269	9.57
16	1.84 × 10^17^	238	259	8.94
17	3.12 × 10^17^	242	263	8.66
18	2.16 × 10^17^	240	260	8.38
19	3.18 × 10^17^	244	263	7.97
20	2.23 × 10^19^	270	290	7.44
21	3.73 × 10^19^	274	262	4.53
22	2.59 × 10^19^	274	260	5.08
23	3.06 × 10^18^	263	249	5.43
24	1.17 × 10^19^	275	286	4.11
25	1.45 × 10^20^	296	313	5.75
26	3.25 × 10^20^	312	308	1.18
27	2.88 × 10^21^	346	326	5.87
28	1.12 × 10^24^	423	424	0.15

**Figure 15:**
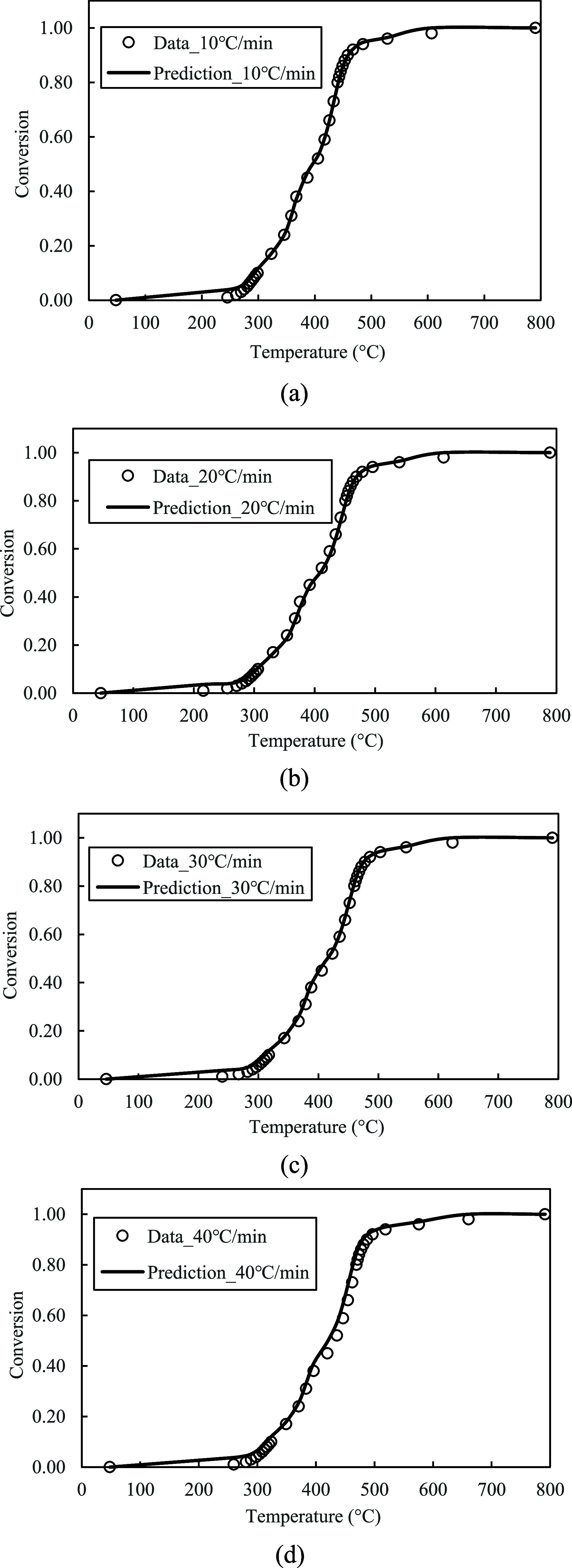
(a–d): Model predictions of α at 10, 20, 30, and 40 °C/min.

**Figure 16:**
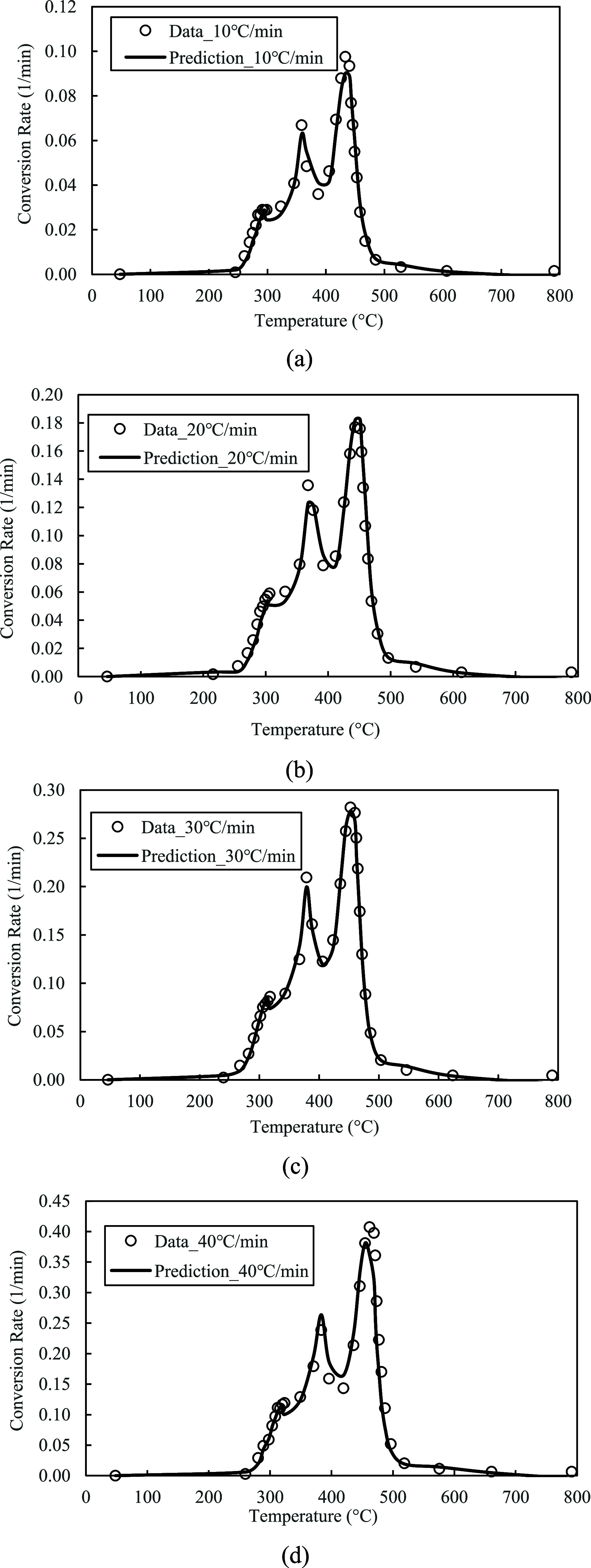
(a–d): Model predictions of $\frac{d\alpha }{dt}$ at 10, 20, 30, and 40 °C/min.

To examine the model accuracy quantitatively, the *R*-squared values were calculated for both the predictions of α and $\frac{d\alpha }{dt}$ at all four heating rates. As listed in Table 2, the predictions of α demonstrated a perfect model fit, with the *R*-squared values above 0.99 for the predicted α at all four heating rates. Moreover, the *R*-squared values for the predictions of $\frac{d\alpha }{dt}$ , a feature very sensitive to temperature changes, were in the range of 0.9233–0.9822, indicating an excellent model fit.

**Table 2:** *R* ^2^ Values of the Kinetic Model in Module 1

heating rate (°C/min)	*R* ^2^ for the conversion **α**	*R* ^2^ for the rate $\frac{d\alpha }{dt}$
10	0.9988	0.9822
20	0.9991	0.9825
30	0.9984	0.9939
40	0.9930	0.9233

On the other hand, a high *R*-squared value may indicate a good model fit but does not guarantee that the model is structured properly. To further assess the model accuracy and ensure a proper structure of the kinetic model, the residuals between the experimental data and the predictions of $\frac{d\alpha }{dt}$ were calculated at all four heating rates. As shown in Figure 17a–d, the residuals from the predicted $\frac{d\alpha }{dt}$ were randomly scattered around the zero axis. There were no visible trends of the residuals, and the residuals showed a roughly constant spread. This indicates that the residuals are random and unbiased, confirming that the model is well-structured.

**Figure 17:**
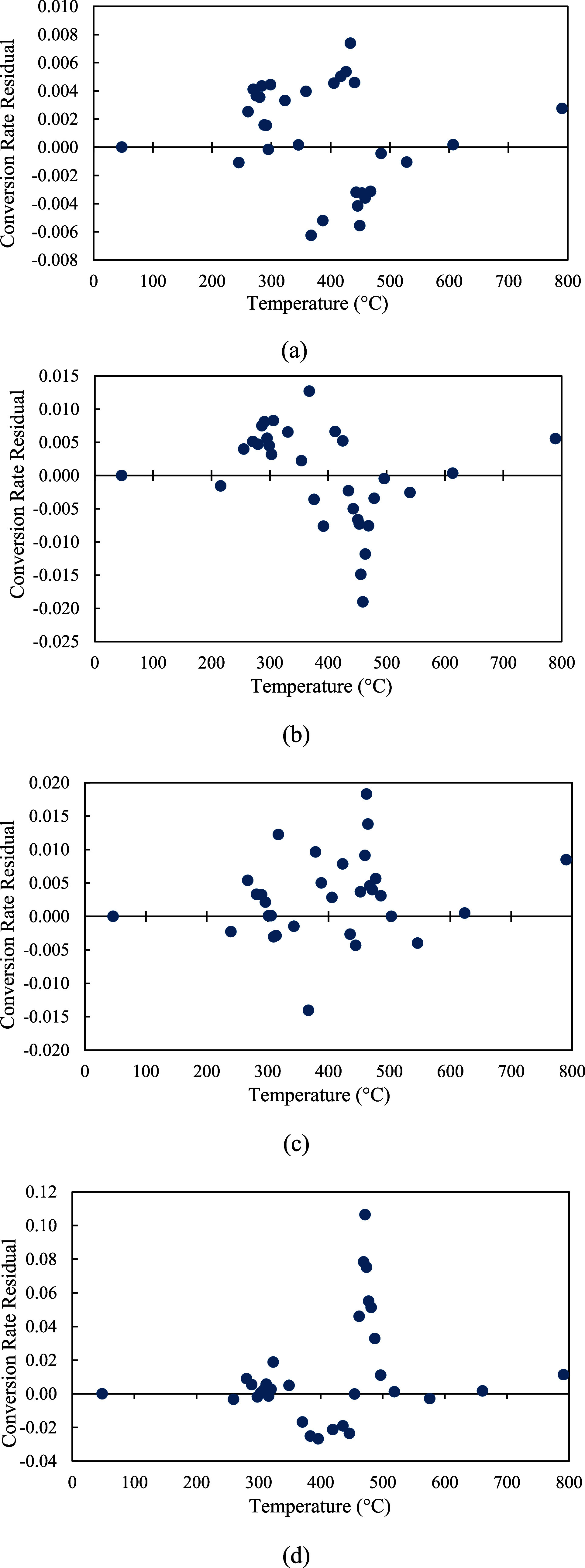
Residual plots of $\frac{d\alpha }{dt}$ at 10 °C/min (a), 20 °C/min (b), 30 °C/min (c), and 40 °C/min (d).

Additionally, in this work, 28 reaction steps were chosen in the parallel reaction mechanism, based on the data-fitting results. Due to a high number of reaction steps, there was concern about overparameterization or model overfitting. To mitigate this concern, the training and validation data sets were separated from the testing data sets to prevent data leakage and ensure robust testing. The kinetic model was trained and validated with the TGA data at 10, 20, and 30 °C/min, while the model performance was subsequently tested with the TGA data at 40 °C/min.

In summary, the model accuracy was thoroughly examined through direct prediction comparisons, evaluation of *R*-squared values, examination of residual scatter patterns, and data set treatment to avoid model overfitting. As a result, the validity of the kinetic model using the parallel-reaction mechanism was fully proven in Module 1.

In Module 2, the experimental data from the vertical-tube reactor were utilized in an optimization model using the SLSQP method to determine the mass distribution coefficients of char, tar, and gas from copyrolysis. As displayed in Table 3, the calculated mass distribution coefficients were assigned to the parallel reaction mechanism in the kinetic model to predict the yields of char, tar, and gas at various temperature settings.

**Table 3:** Coefficients of Char, Tar, and Gas for Module 2

reaction no.	char	tar	gas	reaction no.	char	tar	gas
1	0.2765	0.4886	0.2349	15	0.1282	0.7049	0.1669
2	0.0183	0.9432	0.0384	16	0.0999	0.4358	0.4642
3	0.3249	0.4735	0.2016	17	0.2043	0.7153	0.0804
4	0.1488	0.7347	0.1165	18	0.3562	0.4832	0.1605
5	0.2189	0.6987	0.0824	19	0.1484	0.4966	0.3549
6	0.1035	0.4794	0.4170	20	0.1780	0.5944	0.2277
7	0.3526	0.4559	0.1915	21	0.1652	0.7036	0.1312
8	0.1103	0.4784	0.4113	22	0.3703	0.4398	0.1900
9	0.1277	0.7223	0.1500	23	0.1880	0.6592	0.1527
10	0.1218	0.6108	0.2673	24	0.1914	0.6516	0.1570
11	0.1213	0.7806	0.0981	25	0.3814	0.4550	0.1635
12	0.1279	0.7747	0.0974	26	0.1799	0.7044	0.1157
13	0.2762	0.5173	0.2065	27	0.2694	0.5438	0.1868
14	0.2491	0.5173	0.2336	28	0.4607	0.3042	0.2351

The parallel reaction mechanism using these mass distribution coefficients presented accurate yield predictions of char, tar, and gas for the experiments in the vertical-tube reactor at the temperature settings of 500 and 600 °C. A good agreement between the predictions and experimental data was observed in Figure 18a,b. The prediction errors of char, tar, and gas were 0.34, 0.21, and 0.39% at the temperature setting of 500 °C and were 0.16, 0.21, and 0.21% at the temperature setting of 600 °C. However, as demonstrated in Figure 18c, significant discrepancy was discovered in the predictions at the temperature setting of 700 °C, and the errors were 1.56, 117.01, and 62.04% for char, tar, and gas, respectively. As mentioned in Section 3.3, the large prediction errors were due to the exclusion of tar decomposition from the reaction mechanism in Module 2, which is why the tar decomposition needs to be considered in Module 3 to finish the modeling work.

**Figure 18:**
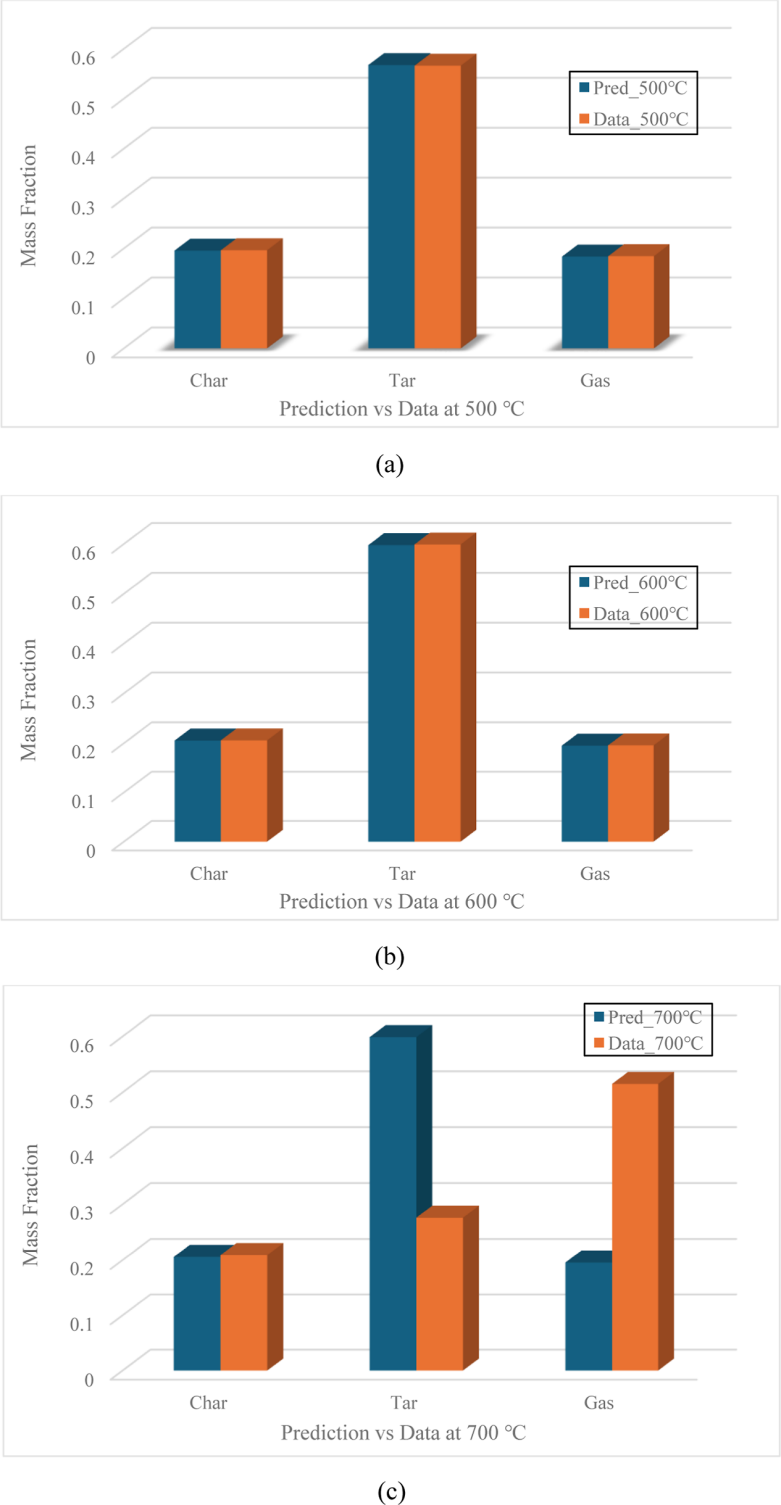
(a–c): Prediction vs experimental data for char, tar, and gas in module 2.

It should be noted that copyrolysis experiments in the vertical-tube reactor were nonisothermal. As described previously, in each experiment, the solid mixture was heated at a constant heating rate of 10 °C/min. After reaching the target temperature, the mixture sample was held at that temperature for 1 h to ensure complete decomposition of the mixture. The experimental temperature settings of 500, 600, and 700 °C were referred to the maximum or target reactor temperatures in each experiment.

In Module 3, the kinetic model was restructured by adding tar decomposition reactions to the parallel reaction mechanism. An optimization model using the PSO algorithm was developed to calculate the optimal activation energies for each tar decomposition reaction. An initial activation energy of 315 kJ/mol was used to initiate the optimization process. During the computation, the activation energies were varied between the lower and upper bounds, which were defined as 0.1 and 1.9 times the initial activation energy, to cover the typical range for tar decomposition. The pre-exponential factors were defined with constant values of 2.23 × 10^23^ s^–1^ to simplify the model computation. The optimal activation energies of tar decomposition for each reaction step were listed in Table 4. The initial values of activation energy and pre-exponential factor were from the work by Fuentes et al.^58^

**Table 4:** Activation Energy (*E*_a_) of Tar Decomposition from Module 3

reaction no.	*E*_a_ (kJ/mol)	reaction no.	*E*_a_ (kJ/mol)
1	392.33	15	384.54
2	278.19	16	280.89
3	279.23	17	279.08
4	276.56	18	281.87
5	496.43	19	280.54
6	277.93	20	277.89
7	569.47	21	278.18
8	419.44	22	540.39
9	277.82	23	277.62
10	279.49	24	282.33
11	277.29	25	279.01
12	446.76	26	358.84
13	503.97	27	277.02
14	277.33	28	280.06

The kinetic model using the combined reaction mechanism was utilized to simulate the transient process of copyrolysis in the vertical-tube reactor at the temperature settings of 500, 600, and 700 °C. The predicted transient profiles of char, tar, gas, and the mixture of red oak wood and PET were shown in Figure 19a–c. As predicted by the kinetic model, the solid mixture of red oak wood and PET began to decompose as the reactor temperature increased. The solid decomposition was initially slow in the period of 40 min, then increased significantly, and finally stopped after the reactor temperatures reached the target temperatures. Meanwhile, char content increased gradually and then remained stable after reaching its maximum. This trend in char content was observed for all three experiments. Tar and syngas exhibited similar trends in the experiments at the settings of 500 and 600 °C, increasing at the beginning and remaining constant afterward. However, the trends of tar and syngas in the experiments at 700 °C were different. During the experiment, tar content decreased quickly after reaching its maximum, while syngas content increased significantly. The simultaneous decrease in tar and increase in syngas was due to the decomposition of tar when the reaction temperature exceeded 600 °C. The predicted trends in tar and syngas were consistent with the experimental results for the copyrolysis in the tube furnace.

**Figure 19:**
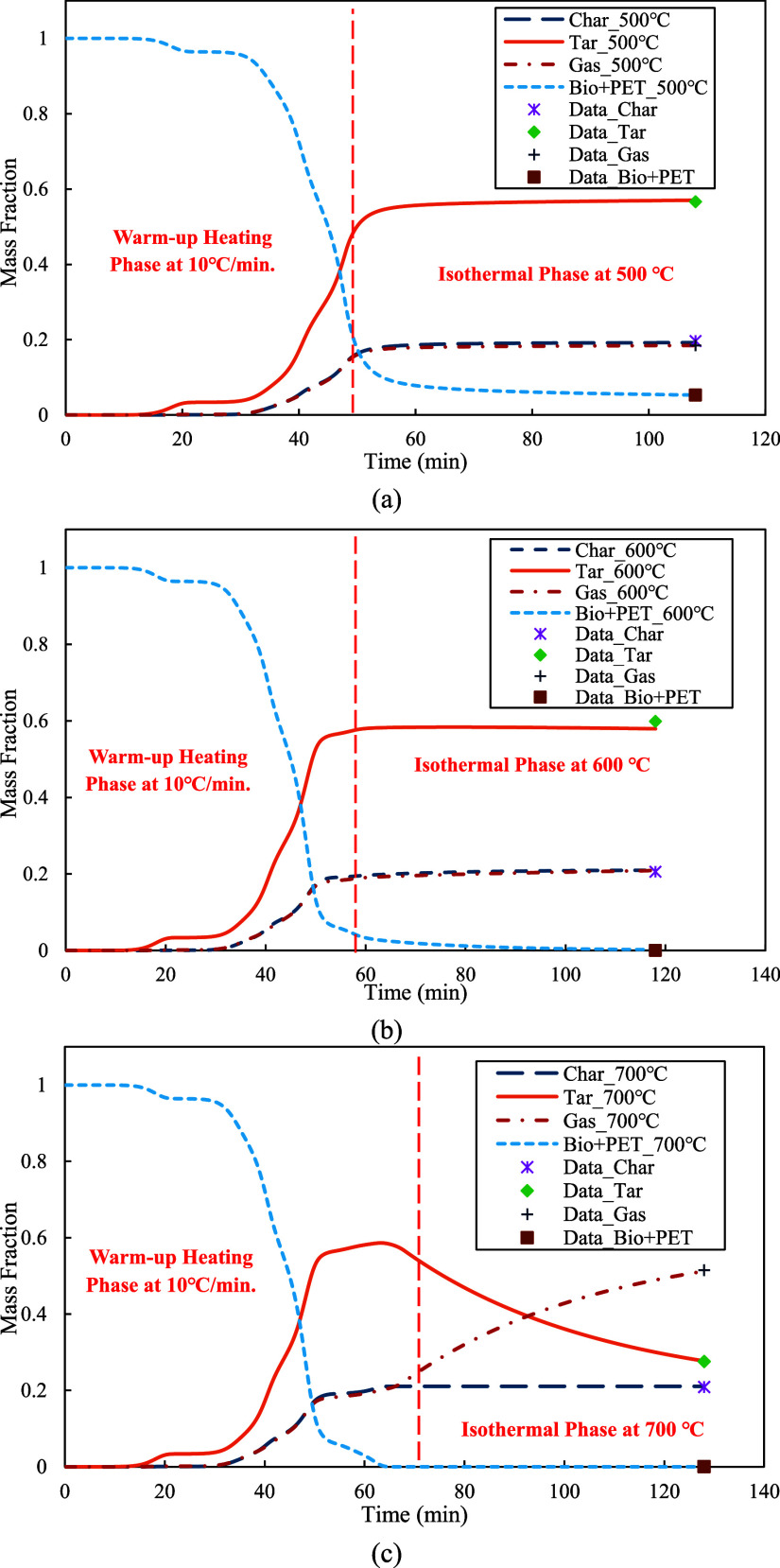
(a–c): The predicted transient profiles of char, tar, gas, and bio & PET in module 3.

To further examine the model accuracy, the yield predictions of char, tar, and gas were compared with experimental data at three temperature settings of 500, 600, and 700 °C. As demonstrated in Figure 20a–c, all the yield predictions closely matched the experimental data at all three temperature settings. The prediction errors of char, tar, and gas were 2.17, 0.69, and 0.06% at the temperature setting of 500 °C, 1.98, 3.26, and 6.87% at the temperature setting of 600 °C, and 0.81, 0.40, and 0.54% at the temperature setting of 700 °C, which indicated an excellent model fit and fully validated the kinetic model using the combined reaction mechanism.

**Figure 20:**
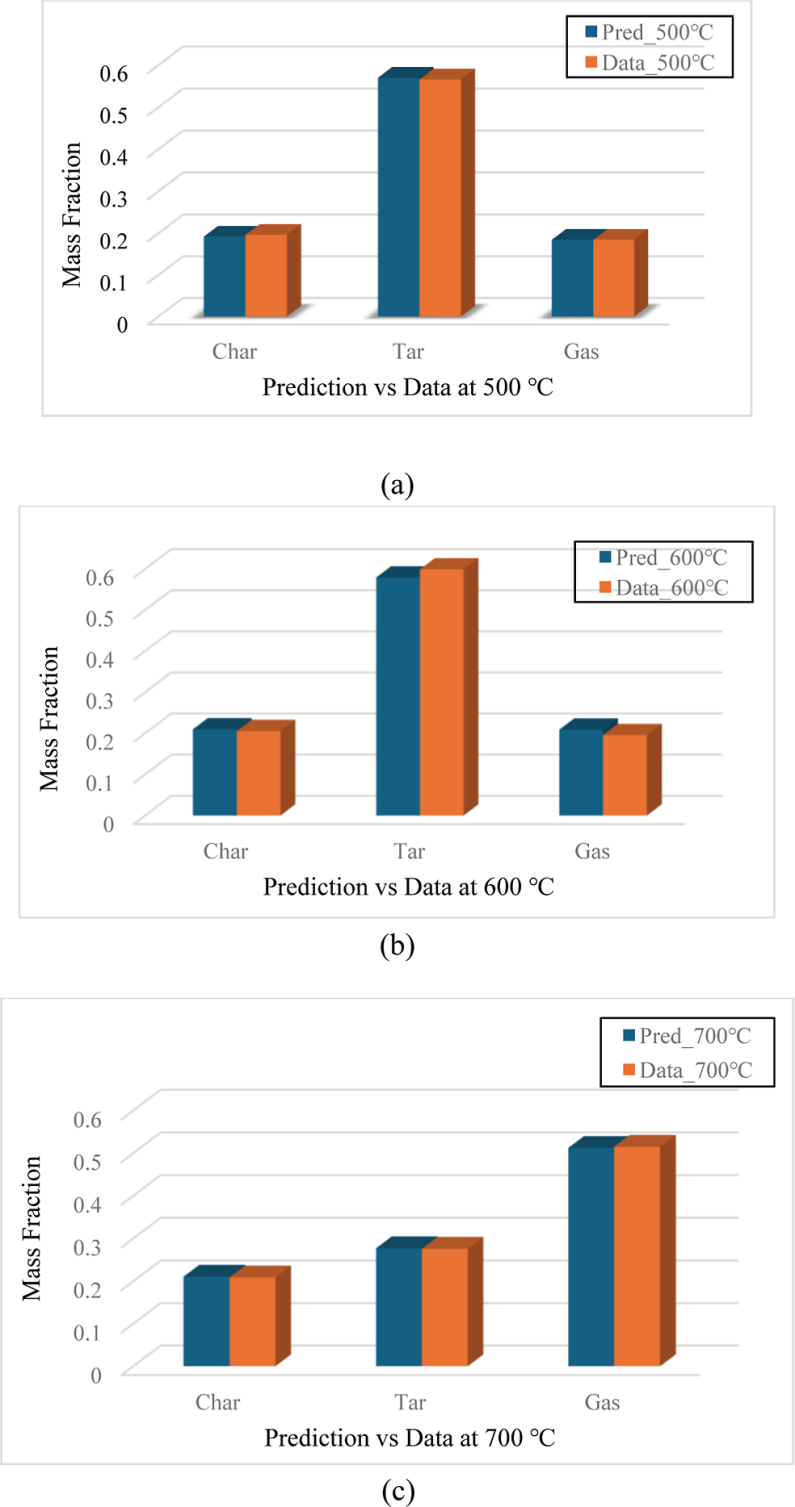
(a–c): Prediction vs experimental data for char, tar, and gas in module 3.

## 5 Conclusions

The modeling work of copyrolysis is always challenging, due to the complex properties of biomass and plastics. Furthermore, exploring an appropriate reaction mechanism and finding proper kinetic parameters make the modeling more difficult. This study aims to provide a systematic approach to address these issues.

In this study, a modeling framework was developed to present an approach for simulating the copyrolysis of red oak wood and PET at a 1:1 mixing ratio. The framework consisted of three modules. In Module 1, a parallel-reaction mechanism was developed, and the kinetic parameters for this mechanism were initially estimated using the Friedman method and then refined through a least-squares optimization model. The parallel-reaction mechanism using the optimal kinetic parameters was applied in a kinetic model to simulate copyrolysis. Since the kinetic model was solely trained with TGA data, it could only predict the conversion of the solid mixture sample and could not provide predictions of bioproducts from copyrolysis.

To solve this problem, in Module 2, the kinetic model was retrained with another type of data, experimental data of copyrolysis in a vertical-tube reactor at various temperature settings. These experimental data were used in an optimization model to calculate the mass distribution coefficients of char, tar, and gas. Subsequently, the parallel reaction mechanism, updated with the optimal coefficients of bioproducts, was used in the kinetic model to simulate copyrolysis. However, due to the exclusion of tar decomposition from the parallel reaction mechanism, the kinetic model did not provide accurate predictions at higher temperature settings, such as 700 °C.

To address this issue, in Module 3, the kinetic model was restructured by adding tar decomposition reactions to the parallel reaction mechanism. The kinetic model was then applied to simulate copyrolysis at various temperature settings. The model was finally validated with experimental data at all three temperature settings (500, 600, and 700 °C).

Overall, the proposed modeling framework was proved to be valid, and the kinetic model provided accurate predictions for both solid conversion and product generation from copyrolysis. The findings and validation suggest that the techniques used to find reaction mechanisms and identify kinetic parameters in this study can be valuable for modeling other pyrolytic processes.

On the other hand, the current modeling approach still needs further improvement. As mentioned earlier, atomic balances were not considered in this modeling work but should be included in future studies when detailed analysis data of tar are available. Additionally, the “lumped” products, such as char, tar, and gas, can be replaced with specific chemicals to allow the kinetic model to provide more valuable information. In this way, the model can be more useful for the design and optimization of pyrolysis reactors. Other future research, such as a CFD simulation of the copyrolysis reactor, can also be pursued to achieve more accurate predictions by accounting for the effects of particle size, momentum transport, heat transfer, and external and internal mass diffusion.

## #

Dr. Hesham Alhumade currently works at King Abdulaziz University, Jeddah, Saudi Arabia

## Nomenclature Symbol Description

α: solid material conversion
*m*_0_: initial mass of solid sample (mg)
*m*_f_: final mass of solid sample (mg)
*m*: mass of solid sample at the current time (mg)
*n*: the number of parallel-reaction steps
*f*(α): reaction mechanism function
$\frac{d\alpha }{dt}$ and *r*: conversion rate
*A*: pre-exponential factor (min^–1^)
*E*_a_: activation energy (kJ/mol)
*T*: reactor temperature (*K*)
*k*_i_: reaction rate constant for the *i*thparallel reaction step
*k*_*tar*,*i*_^′^: reaction rate constant for tar decomposition in the *i*th reaction step
*n*_nm_: mass distribution coefficient of species *m* in the *n*th parallel reaction step
*n*_char,i_: mass distribution coefficient of Char in the *i*th parallel reaction step
*n*_tar,i_: mass distribution coefficient of Tar in the *i*th parallel reaction step
*n*_gas,i_: mass distribution coefficient of Gas in the *i*th parallel reaction step
Char_n_: char generated in the *n*th reaction step
Tar_n_: tar generated in the *n*th reaction step
Gas_n_: gas generated in the *n*th reaction step
Product_nm_: product m in the *n*th parallel reaction step
rate_redoak_: decomposition rate of the red oak sample
rate_PET_: decomposition rate of the PET sample
rate_calulated_: theoretical or calculated decomposition rate of the mixture sample
η_redoak_: mass fraction of red oak in the mixture
η_PET_: mass fraction of PET in the mixture

## References

[1] Ankrah I. (2025). A spotlight on fossil fuel lobby and energy transition possibilities in emerging oil-producing economies. Heliyon.

[2] Abbasi T., Abbasi S. A. (2011). Decarbonization of fossil fuels as a strategy to control global warming. Renew. Sustain. Energy Rev..

[3] Chiari L., Zecca A. (2011). Constraints of fossil fuels depletion on global warming projections. Energy Policy.

[4] Dahal U. (2024). Green dreams, local realities: Complexities of the European Union’s energy transition to ensure local health and well-being in a fossil fuel-based industrial region. Environ. Impact Assess. Rev..

[5] Zandi Lak, S. ; Rezaei, J. ; Rahimpour, M. R. Health and Pollution Challenges of Fossil Fuels Utilization. In Encyclopedia of Renewable Energy, Sustainability and the Environment, First ed. ed.; Rahimpour, M. R. , Ed.; Elsevier: Oxford, 2024; pp 155–166.

[6] Liu Y., Huang Y. (2024). Assessing the interrelationship between fossil fuels resources and the biomass energy market for achieving a sustainable and green economy. Resour. Policy.

[7] Yao S., Li T., Li Y. (2023). Promoting sustainable fossil fuels resources in BRICS countries: Evaluating green policies and driving renewable energy development. Resour. Policy.

[8] Bays H. C. M. (2024). Assessing the sustainability of forest biomass harvesting practices in the southeastern US to meet European renewable energy goals. Biomass and Bioenergy.

[9] Nawaz A. (2024). Fueling sustainability: Co-pyrolysis of microalgae biomass and waste plastics for renewable energy and waste mitigation. Biomass and Bioenergy.

[10] Li P. (2025). Research progress on the preparation of high-value carbon materials by biomass pyrolysis. Biomass and Bioenergy.

[11] Liu N. (2025). Effects of pretreatment procedure, compositional feature and reaction condition on the devolatilization characteristics of biomass during pyrolysis process: A review. J. Energy Inst..

[12] Ma C. (2025). Kinetics, reaction mechanism and product distribution of lignocellulosic biomass pyrolysis using triple-parallel reaction model, combined kinetics, Py-GC/MS, and artificial neural networks. Ind. Crops Prod..

[13] Cupertino G. F. M. (2024). Co-pyrolysis of biomass and polyethylene terephthalate (PET) as an alternative for energy production from waste valorization. Fuel.

[14] Ojha, D. K. , Vinu, R. , Chapter 12—Copyrolysis of Lignocellulosic Biomass With Waste Plastics for Resource Recovery, in Waste Biorefinery, Bhaskar, T. , etal, Eds. 2018, Elsevier. p 349–391.

[15] Patra, B. R. , , Chapter 5—Advances in pyrolysis and copyrolysis technologies for biomass conversion to solid and liquid biofuels, in Biomass to Bioenergy, Nanda, S. ; Dalai, A. K. , Eds. 2024, Woodhead Publishing. p 109–126.

[16] Chen Y. (2024). In-depth understanding of the synergistic effect in catalytic copyrolysis of lignin-plastic mixtures with lignin-tailored hierarchical HZSM-5 catalysts. Fuel.

[17] Hong Y. (2017). In-situ catalytic copyrolysis of cellulose and polypropylene over desilicated ZSM-5. Catal. Today.

[18] Wang S. (2021). Synergistic effects in the copyrolysis of municipal sewage sludge digestate and salix: Reaction mechanism, product characterization and char stability. Appl. Energy.

[19] Bilad, M. R. ; Abu Bakar, M. S. ; Usman, A. 6—Bio-oil production from waste and waste plastics. In Waste Valorization for Bioenergy and Bioproducts; Ong, H. C. , Fattah, I. M. R. , Mahlia, T. M. I. , Eds.; Woodhead Publishing, 2024; pp 121–138.

[20] Lopes F. C. R., Tannous K. (2020). Coconut fiber pyrolysis decomposition kinetics applying single- and multi-step reaction models. Thermochim. Acta.

[21] Sangaré D. (2024). Multi-step kinetic mechanism coupled with CFD modeling of slow pyrolysis of biomass at different heating rates. Chem. Eng. J..

[22] Shen T. (2025). Pyrolysis behavior and kinetics of grape pruning residues: Insights from multistep reaction models and Py-GC/MS. J. Anal. Appl. Pyrolysis.

[23] Vo T. A. (2021). Lumped-kinetic modeling and experiments on co-pyrolysis of palm kernel cake with polystyrene using a closed-tubing reactor to upgrade pyrolysis products. Energy Convers. Manage..

[24] Safavi A., Richter C., Unnthorsson R. (2024). A Study of Parallel and Competitive Reaction Schemes in Kinetic Modeling of Plastic Pyrolysis. ACS Omega.

[25] Lorbach S.-M. (2024). Usefulness of Lumped Kinetic Modeling. Chem. & Bio Eng..

[26] Ganesan A. (2025). Kinetic Analysis of Construction, Renovation, and Demolition (CRD) Wood Pyrolysis Using Model-Fitting and Model-Free Methods via Thermogravimetric Analysis. Energies.

[27] Ismail M. A. (2025). Assessing Lemon Peel Waste as a Solid Biofuel: A Study of Its Combustion Behaviour, Kinetics, and Thermodynamics. Polymers.

[28] Silva J. P., Teixeira S., Teixeira J. C. (2025). Thermogravimetric Assessment and Kinetic Analysis of Forestry Residues Combustion. Energies.

[29] Torres-Sciancalepore R. (2022). Kinetic and thermodynamic comparative study of quince bio-waste slow pyrolysis before and after sustainable recovery of pectin compounds. Energy Convers. Manage..

[30] Torres-Sciancalepore R. (2023). Two-step valorization of invasive species Rosa rubiginosa L. husk waste through eco-friendly optimized pectin extraction and subsequent pyrolysis. J. Environ. Chem. Eng..

[31] Torres-Sciancalepore R. (2024). Synergistic effects of the mixing factor on the kinetics and products obtained by co-pyrolysis of Rosa rubiginosa rosehip seed and husk wastes. Energy Convers. Manage..

[32] Jain A. A., Mehra A., Ranade V. V. (2016). Processing of TGA data: Analysis of isoconversional and model fitting methods. Fuel.

[33] Li M. (2023). The impact of heating rate on the decomposition kinetics and product distribution of algal waste pyrolysis with in-situ weight measurement. Chem. Eng. J..

[34] Vyazovkin S. (2020). Kissinger Method in Kinetics of Materials: Things to Beware and Be Aware of. Molecules.

[35] Arenas C. N., Navarro M. V., Martínez J. D. (2019). Pyrolysis kinetics of biomass wastes using isoconversional methods and the distributed activation energy model. Bioresour. Technol..

[36] Guo X., Liu C. (2024). Thermogravimetric analysis on two Bambusa textilis wastes slow pyrolysis behaviors and kinetics using isoconversional and parallel-reactions models. Thermochim. Acta.

[37] Nie N. (2023). Exploring kinetic and thermodynamic mechanisms of switchgrass pyrolysis using iterative linear integral isoconversional method and master plots approach. Fuel.

[38] Alhulaybi Z., Dubdub I. (2023). Comprehensive Kinetic Study of PET Pyrolysis Using TGA. Polymers.

[39] Choi M.-J., Jeong Y.-S., Kim J.-S. (2021). Air gasification of polyethylene terephthalate using a two-stage gasifier with active carbon for the production of H2 and CO. Energy.

[40] Jia H. (2020). Catalytic Fast Pyrolysis of Poly (Ethylene Terephthalate) (PET) with Zeolite and Nickel Chloride. Polymers.

[41] Carrasco J. C., Oporto G. S., Zondlo J., Wang J. (2013). Torrefaction kinetics of red oak (Quercus rubra) in a fluidized reactor. BioRes..

[42] Hu W. (2024). Combustion Behaviors, Kinetics, and Thermodynamics of Naturally Decomposed and Torrefied Northern Red Oak (Quercus rubra) Forest Logging Residue. Energies.

[43] Amrullah A., Farobie O., Septarini S., Satrio J. A. (2022). Synergetic biofuel production from co-pyrolysis of food and plastic waste: reaction kinetics and product behavior. Heliyon.

[44] M A. K., A.P S., J S., C S. D., P S., Hatamleh A. A., Al-Dosary M. A., Mani R. R., Chung W. J., Chang S. W. (2024). Pyrolysis behaviour and synergistic effect in co-pyrolysis of wheat straw and polyethylene terephthalate: A study on product distribution and oil characterization. Heliyon.

[45] Miskolczi N., Gao N. B., Quan C. (2024). Transformation of biomass and waste plastic mixtures into hydrocarbon oils and gases by pyrolysis using different reactor temperatures and pressures. J. Anal. Appl. Pyrolysis.

[46] Seah C. C. (2023). Co-pyrolysis of biomass and plastic: Circularity of wastes and comprehensive review of synergistic mechanism. Results Eng..

[47] Komandur J. (2022). Kinetic Parameters Estimation of Thermal and Co-Pyrolysis of Groundnut De-oiled Cake and Polyethylene Terephthalate (PET) Waste. Energies.

[48] Kumar Mishra R., Mohanty K. (2020). Co-pyrolysis of waste biomass and waste plastics (polystyrene and waste nitrile gloves) into renewable fuel and value-added chemicals. Carbon Resour. Convers..

[49] Van de Velden M. (2010). Fundamentals, kinetics and endothermicity of the biomass pyrolysis reaction. Renew. Energy..

[50] Brems A. (2011). Thermogravimetric pyrolysis of waste polyethylene-terephthalate and polystyrene: A critical assessment of kinetics modelling. Resour. Conserv. Recycl..

[51] Liu H., Cattolica R. J., Seiser R. (2016). CFD studies on biomass gasification in a pilot-scale dual fluidized-bed system. Int. J. Hydrogen Energy.

[52] Liu H. (2020). A hybrid kinetic and optimization approach for biomass pyrolysis: The hybrid scheme of the isoconversional methods, DAEM, and a parallel-reaction mechanism. Energy Convers. Manage..

[53] Liu H., Alhumade H., Elkamel A. (2023). A combined scheme of parallel-reaction kinetic model and multi-layer artificial neural network model on pyrolysis of Reed Canary. Chem. Eng. Sci..

[54] Dong Z. (2018). Theoretical Analysis of Double Logistic Distributed Activation Energy Model for Thermal Decomposition Kinetics of Solid Fuels. Ind. Eng. Chem. Res..

[55] Miura K. (2024). Pseudo Master Curve Analysis of an Infinite Number of Parallel First-Order Reactions: Improved Distributed Activation Energy Model. ACS Omega.

[56] Wu S. (2024). A Discrete Distributed Activation Energy Model for Cedar and Polyethylene Fast Heating Pyrolysis Kinetics. Processes.

[57] Wu F. (2020). Effects of Different Conditions on Co-Pyrolysis Behavior of Corn Stover and Polypropylene. Polymers.

[58] Fuentes-Cano D. (2016). Kinetic Modeling of Tar and Light Hydrocarbons during the Thermal Conversion of Biomass. Energy Fuels.

